# X-ray Fluorescence Microscopy to Develop Elemental Classifiers and Investigate Elemental Signatures in BALB/c Mouse Intestine a Week after Exposure to 8 Gy of Gamma Rays

**DOI:** 10.3390/ijms251910256

**Published:** 2024-09-24

**Authors:** Anthony Smith, Katrina Dobinda, Si Chen, Peter Zieba, Tatjana Paunesku, Zequn Sun, Gayle E. Woloschak

**Affiliations:** 1 Feinberg School of Medicine, Northwestern University, Chicago, IL 60611, USA; 2 X-ray Imaging Division, Advanced Photon Source, Argonne National Laboratory, Argonne, IL 60439, USA

**Keywords:** X-ray fluorescence microscopy, gastrointestinal acute radiation syndrome, elemental cell classifiers

## Abstract

Iron redistribution in the intestine after total body irradiation is an established phenomenon. However, in the literature, there are no reports about the use of X-ray fluorescence microscopy or equivalent techniques to generate semi-quantitative 2D maps of iron in sectioned intestine samples from irradiated mice. In this work, we used X-ray fluorescence microscopy (XFM) to map the elemental content of iron as well as phosphorus, sulfur, calcium, copper and zinc in tissue sections of the small intestine from eight-week-old BALB/c male mice that developed gastrointestinal acute radiation syndrome (GI-ARS) in response to exposure to 8 Gray of gamma rays. Seven days after irradiation, we found that the majority of the iron is localized as hot spots in the intercellular regions of the area surrounding crypts and stretching between the outer perimeter of the intestine and the surface cell layer of villi. In addition, this study represents our current efforts to develop elemental cell classifiers that could be used for the automated generation of regions of interest for analyses of X-ray fluorescence maps. Once developed, such a tool will be instrumental for studies of effects of radiation and other toxicants on the elemental content in cells and tissues. While XFM studies cannot be conducted on living organisms, it is possible to envision future scenarios where XFM imaging of single cells sloughed from the human (or rodent) intestine could be used to follow up on the progression of GI-ARS.

## 1. Introduction

Exposure to total body irradiation at high doses of ionizing radiation, such as 8 Gy of gamma rays, causes lethal gastrointestinal acute radiation syndrome (GI-ARS) in mice [1,2,3]—a condition associated with cell death of the intestine replenishing cells in the intestinal crypts. This cell death is not only caused by DNA damage at the time of radiation injury but also crypt cells continue to die for days after radiation exposure has concluded. Different mechanisms of cell death have been proposed as a probable cause of death, including ferroptosis [2,4,5,6]. Following irradiation, an increase in bulk iron content was noted in mice exposed to 5 or 10 Gy at 1, 3 and 7 days after exposure [2,5]. In the Göttingen Minipig model, increased ferritin content was noted even in the intestines of animals exposed to doses causing subacute injury to the GI tract [4]. In mice and non-human primates, radiation exposure was reported to lead to accumulation of iron not only in the intestine but also in the spleen, bone marrow, liver and heart [6,7]. According to some studies, this iron increase is related to radiation-induced hemolysis of red blood cells [6,7], while others attributed it to a polarization shift of macrophages from the iron-accumulating M1 phenotype to the iron-releasing M2-like phenotype [8]. In the intestines of irradiated mice, an increase in iron content was associated with an increase in ferroptosis [2,5,6]. Considering that the iron accumulation leading to ferroptosis occurs over an extended period after radiation injury, understanding the cause of the iron increase could help us to develop strategies to reduce crypt cells death and mitigate some of the symptoms of GI-ARS. To that end, it would be particularly useful to begin such an investigation by evaluating iron distribution and its changes over time in different intestinal tissue regions and cell structures at multiple timepoints after radiation exposure. Our laboratory houses an archive of tissues from radiation exposed animals [9,10,11,12], including exposures to external beams as well as internal emitters. While these archival intestinal tissues could provide an unprecedented number of samples, high throughput and automated approaches for analyses will be necessary for such work.

Different approaches for the histological evaluation of intestinal samples have been perfected over the years [13]. However, the distribution of iron in mouse intestine after ionizing radiation exposure was never evaluated by elemental mapping using X-ray fluorescence microscopy (XFM). Most of the studies that discuss iron content have focused either on bulk iron [2,5] or (using Western blots) iron containing proteins such as ferritin [4]. Less frequently, Prussian blue staining and immunohistochemistry (IHC) for iron binding proteins have been performed and then followed by visible light microscopy [6]. It should be noted that Prussian blue detects iron only in the chemical form of Fe^3+^, while IHC recognizes only specific proteins. XFM, on the other hand, reveals the presence of iron in any chemical form, including free iron ions as well as forms associated with iron-binding proteins. For that reason, XFM imaging could reveal all locations of iron in GI tissues and cells and aid in the evaluation of treatments developed for iron management such as the potential use of iron chelators developed for the treatment of thalassemia [14] or Alzheimer’s disease [15].

XFM is a semi-quantitative technique for the evaluation of elemental content in a variety of specimens. For typical data collection, the incident X-ray photons are focused with X-ray optics onto a small spot of a specimen [16,17]. While a certain area of the specimen is raster scanned through the focused beam, complete fluorescence spectra are acquired for each step of a scan. Each X-ray spectrum acquired in this manner corresponds to a single pixel of a resultant elemental map of the area of the specimen that is scanned. When the beam focal size is larger than the scan step size (pixel size), this is referred to as “oversampling”, while “undersampling” is the opposite. Both are referred to as sampling density. Usually, slight oversampling is employed for data collection to ensure that the area of interest is completely exposed to X-rays without any gaps. In that case, the spatial resolution of the resulting elemental maps is mainly determined by the focal size of the X-ray beam. However, undersampling is also used in some situations in order to rapidly obtain an overview of a relatively large area. In these images, the elemental content of each pixel is represented by the measurement of those sub-regions of the sample where the X-ray beam impinges on the specimen.

In this study, we conducted XFM with the Bionanoprobe instrument [18,19,20] at the Advanced Photon Source (APS) at Argonne National Lab (ANL). We imaged small intestine tissue samples from irradiated mice and their control counterparts. Imaging was undertaken using a highly focused X-ray beam of sub-100 nm at full width at half maximum (FWHM), which permitted us to determine whether iron was present inside cells or in the inter-cellular region. The step size used for scanning the specimens varied from 100 nm, matching the beam size, to several times greater than the beam. We used MAPS software (https://www.aps.anl.gov/Microscopy/Software-and-Tools-MAPS accessed on 10 August 2024) as we did in the past for the evaluation of elemental content in animal tissues and single cells [21,22,23,24,25,26,27] and conducted region of interest analyses. In some cases, the regions of interest (ROI) were outlined based on tissue regions—areas of individual crypts and areas surrounding them. We found that the foci with the highest iron concentration occurred most frequently in the tissue regions surrounding crypts and located below the surface cell layer of villi. In this paper, this tissue region is referred to as “interspersed cell region”. Uniform Manifold Approximation and Projection (UMAP) analyses of crypt and interspersed cells tissue regions generated graphs that displayed elemental concentration co-distribution for individual XFM map pixels.

In this work, we also conducted extensive ROI analyses of subcellular regions—nuclei and cytosol of individual cells of different types. This was possible because high resolution scanning generated elemental maps with high data density. Each subcellular region ROI area had more than 500 pixels, while many had several thousand pixels. With regard to X-ray fluorescence imaging of identical regions of identical cells using different sampling density, we found that the elemental concentrations in collections of generated datapoints/pixels were statistically significantly different. Thus, we found that sample heterogeneity coupled with sampling differences prevented us from determining the identity of same cells using their elemental content alone. Similarly, cells of the same cell types could not be grouped together into the same categories when we used standard statistical methods. New approaches for data analyses will be required in order to provide automated cell-type categorization based on elemental content. Only with such tools will we be able to benefit fully from XFM elemental analysis and identify different cellular structures and intercellular regions in complex tissue samples using automated approaches.

## 2. Results

### 2.1. Elemental Maps of Control and Irradiated Mouse Intestine Samples

Two pairs of small intestine samples were obtained from BALB/c mice; control animal samples are labeled as noIR-1 and 2, irradiated animal samples as IR-1 and 2 throughout the text. Radiation exposure to 8 Gy of gamma rays caused GI-ARS in irradiated animals and small intestine tissue was collected a week after exposure. Radiation-related tissue changes in these samples were evaluated by visible light microscopy in an earlier study [3].

**Figure 1:**
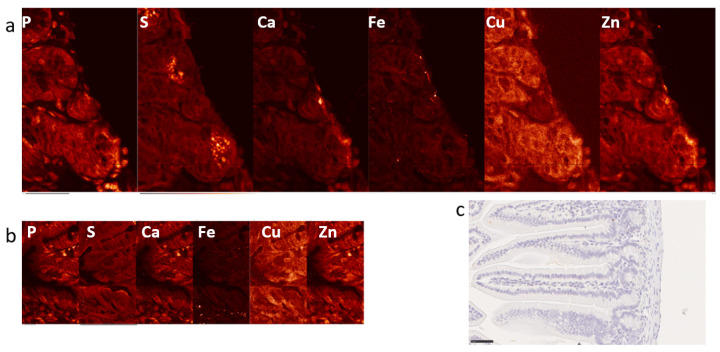
Elemental maps of intestine of the sham irradiated mouse noIR-1 scanned with ~100 nm X-ray beam. Two different areas of the intestine are presented (**a**,**b**) and a visible light image of a similar area of the intestine—IHC staining for macrophage marker F4/80 (**c**). White letters indicate 2D maps of individual elements. Note that P map shows distribution of cellular material, with the strongest P signals in cell nuclei, corresponding with blue hematoxylin staining in (**c**). Scan step sizes were 100 nm and scan areas in *x* and *y* direction were 53 by 84 and 47 by 84 micron. Scale bars in (**a**,**b**) indicate 20 and 10 microns, respectively, and scale bar in (**c**) shows 250 microns.

**Figure 2:**
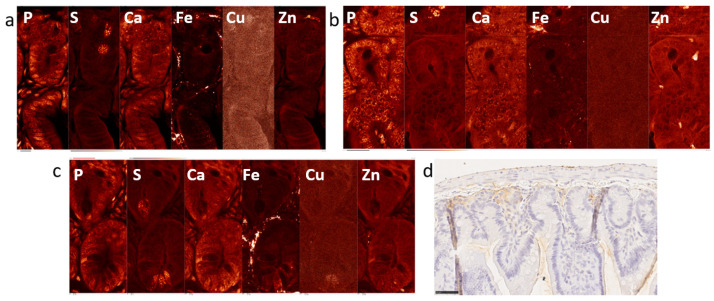
Elemental maps of intestine of the irradiated mouse IR-1 scanned with ~100 nm X-ray beam. Three different areas of the intestine are presented (**a**–**c**) scanned with 100 nm beam step size and covering areas of 48 by 134, 52 by 120, and 54 by 126 micron, respectively. A visible light image of a similar area of the intestine—IHC staining for macrophage marker F4/80 is shown in (**d**). White letters indicate 2D maps of individual elements. Scan step sizes were 100 nm and scan areas in *x* and *y* were 48 by 134, 52 by 120 and 54 by 126 micron. Scale bars in (**a**–**c**) indicate 10 microns, and the scale bar in (**d**) 250 microns.

This work presents findings of XFM conducted on these samples and explores different approaches for data analyses. XFM was conducted using X-ray beam spot size of ~100 nm, but different step scan sizes were used for different images, as indicated in figure legends. Control mouse samples are shown in Figure 1 and Supplementary Figure S1, and XFM images of irradiated intestine samples are presented in Figure 2 and Supplementary Figure S2.

**Figure 3:**
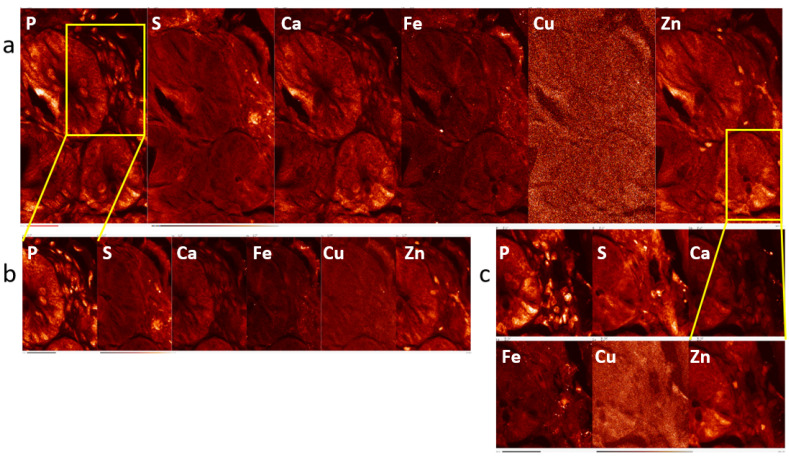
Elemental maps of intestine of the irradiated mouse IR-2 scanned with different step sizes and the same beam spot size. Scanning was performed with ~100 nm X-ray beam, with step size of 250 nm (**a**) or 100 nm (**b**,**c**). White letters indicate 2D maps of individual elements. Yellow rectangles show the sub-regions of a large-step undersampling scan (**a**) that were subsequently imaged with smaller step sizes generating elemental maps in (**b**,**c**). While it is difficult to note differences between these scans for an abundant element such as phosphorus, data density produced with smaller step size scanning is far greater and for elements with low concentrations such as copper (fifth panel in each image), this leads to much better image definition. Scan area sizes were 80 by 136, 52 by 79 and 50 by 56 microns in *x* and *y* for scans (**a**), (**b**) and (**c**), respectively. Scale bars in (**a**–**c**) indicate 20 microns.

In addition, this XFM evaluation also included imaging of the same sample with the same X-ray focus size but with the different scan step sizes—i.e., different degrees of sampling. Figure 3 shows the images obtained for the irradiated mouse IR-2 generated with different step sizes.

### 2.2. Statistical Analyses of ROI Data Extracted from XFM Images

**Figure 4:**
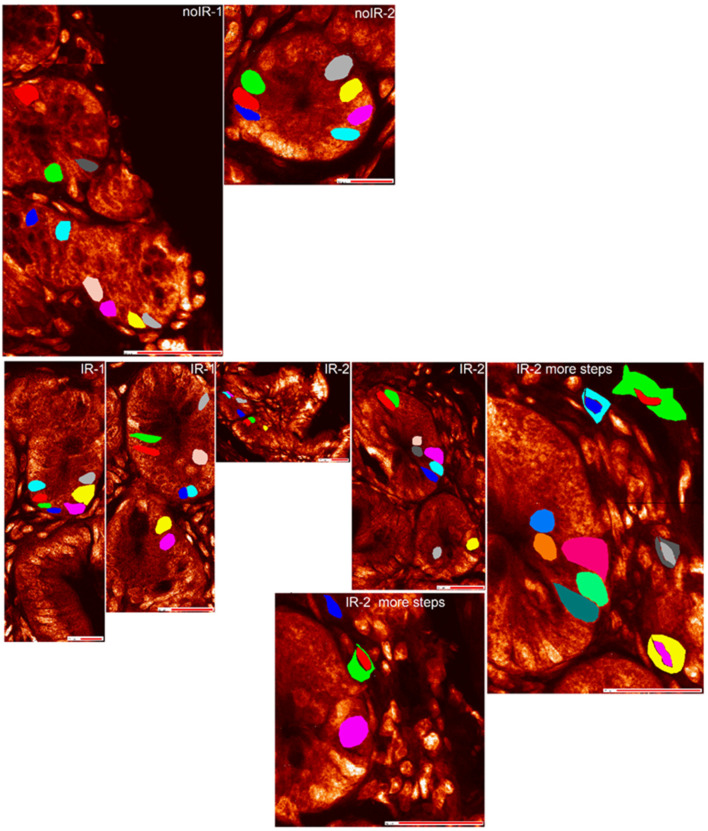
Phosphorus maps of scans used for this work, with ROIs for crypt cell nuclei marked by colors generated by MAPS program (red, green, navy, turquoise, magenta, yellow, gray, dark gray, tan, orange, light green, dark green). For the IR-2 animal where scans were repeated at different sampling densities, regular scan images are indicated by “more steps” added to sample identification. Please note that the ROI colors do not match between IR-2 and IR-2 more steps images. However, we did not use ROI color as cell label after we extracted the scatter plot pixel spectrum data. Instead, the pixel data were associated with specific cell IDs. Scale bar data is provided in phosphorus maps without labeled ROIs in Figure 1, Figure 2 and Figure 3 and Supplemental Figures S1 and S2.

**Figure 5:**
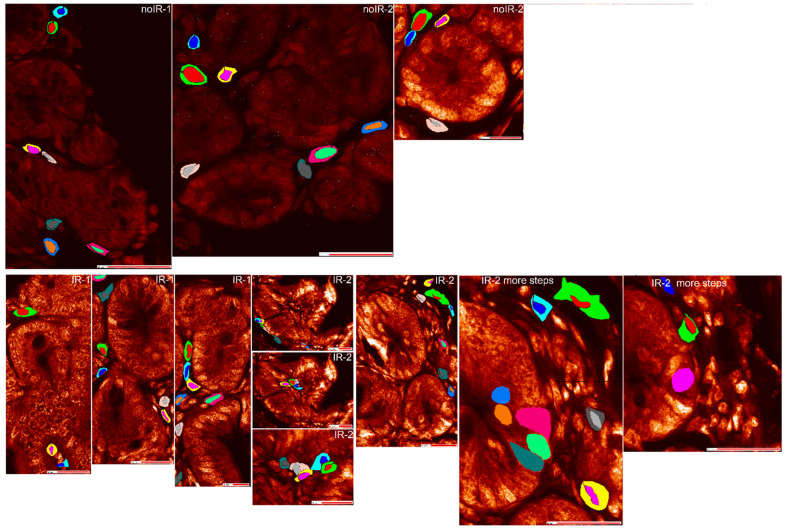
Phosphorus maps of all scans used for this work, with interspersed cell nuclei and cytosol ROIs marked by colors generated by MAPS program (red, green, navy, turquoise, magenta, yellow, gray, dark gray, tan, orange, light green, dark green). For the IR-2 animal where scans were repeated at different sampling densities, regular scan images are indicated by “more steps” added to sample identification. Again, please note that the ROI colors do not match between IR-2 and IR-2 more steps images. While ROIs were used to extract the spectrum data for each pixel, the pixel data were associated with specific cell IDs before statistical analysis. Scale bar data is provided in phosphorus maps without labeled ROIs in Figure 1, Figure 2 and Figure 3 and Supplemental Figures S1 and S2.

**Figure 6:**
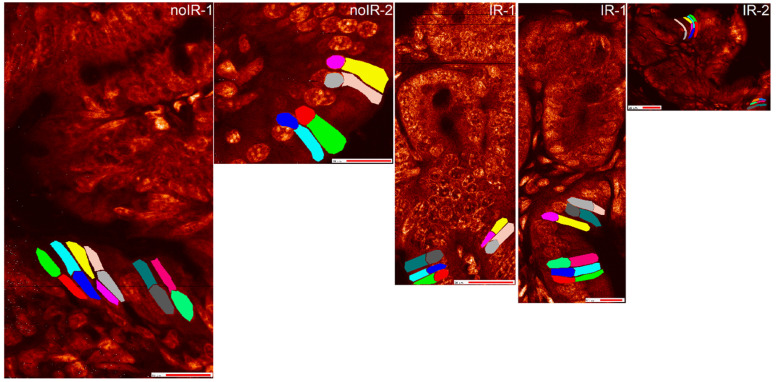
Phosphorus maps of scans used for this work, with villus cell nuclei and cytosol ROIs marked by colors generated by MAPS program (red, green, navy, turquoise, magenta, yellow, gray, dark gray, tan, orange, light green, dark green). Scale bar data is provided in phosphorus maps without labeled ROIs in Figure 1, Figure 2 and Figure 3 and Supplemental Figures S1 and S2.

ROIs were generated for different tissue regions (Supplementary Figures S3–S6) as well as for single-cell nuclei and cytosol regions for different cell types: crypt cells (Figure 4), interspersed cells (Figure 5) and villus cells (Figure 6). In general, two types of elemental information can be obtained from ROI analyses using MAPS software: elemental area content information and elemental individual pixel information. ROI elemental area information shows the cumulative elemental content within the ROI, typically in femtograms. ROI elemental area information also includes information about the size of that area and its mean per pixel elemental concentration expressed in micrograms per centimeter square. On the other hand, ROI elemental individual pixel information provides information about each individual pixel’s elemental concentration expressed in micrograms per centimeter square. Due to the cumbersome interface and slow processing of ROIs with large pixel numbers, the ROI generation and data extraction for analyses shown in this work took many weeks of processing time.

Different types of ROI data analyses were conducted, all with the elemental individual pixel information data. Therefore, all numerical element information in the subsequent part of this manuscript is expressed in micrograms per centimeter squared. It should be noted, however, that in each case, actual area that generated elemental fluorescence corresponds to a circle with ~100 nm diameter—the size of the X-ray beam focus.

**Figure 7:**
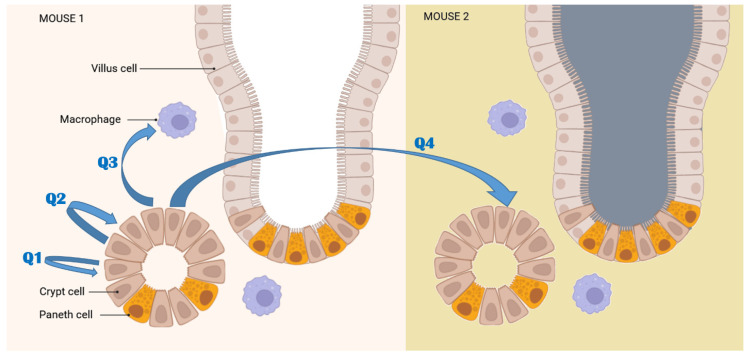
ROI analyses in this work were conducted with the intention to ask four distinct questions. Q1: Will the statistical analysis recognize same subcellular regions from the same cell from two different scans as same? Q2: Will subcellular regions from several cells in the same tissue region be similar? Q3: What will be the level of similarity between subcellular regions from cells from different tissue regions? Q4: What will be the level of similarity between subcellular regions from cells from the equivalent tissue regions in two different animals, especially if one animal was irradiated and the other one was not?

In the interest of generating elemental cell classifiers, we intended to inspect differences between the subcellular regions of cells that were identical but scanned twice and with two distinct ROI analyses, differences between subcellular regions of cells from the same area of the tissue; differences between subcellular regions of cells from a different area of the tissue, or differences between subcellular regions of cells from a similar region in a different animal, as depicted in schematic Figure 7.

#### 2.2.1. Mean Pixel Concentration Analysis—Cell Type Analyses

**Figure 8:**
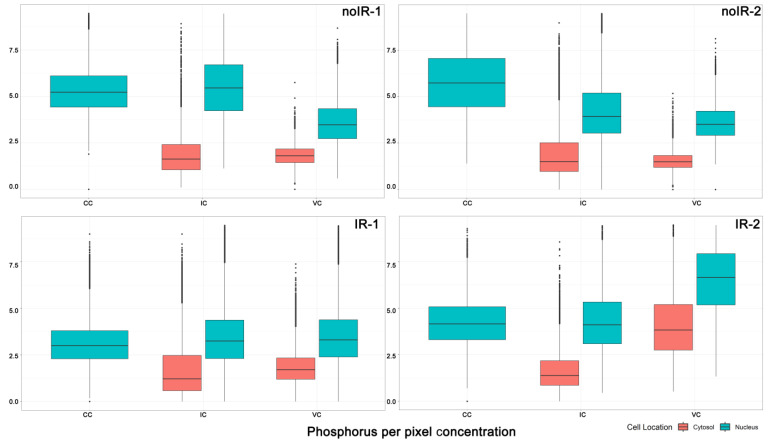
Individual pixel phosphorus concentration for all cell ROIs within a single animal: nuclei = teal and cytosol = pink. The data are grouped into three overarching categories on the *x*-axis: crypt cells, interspersed cells and villus cells. Note that only the nuclei group is represented in crypt cells. For ROI generation, see Figure 4, Figure 5 and Figure 6. For corresponding mean numerical values, see Supplementary Table S1.

Using the simplest approach for evaluation, we calculated the mean value of all individual pixel elemental concentration values from the cytosol portions and nuclei portions of the cells of the same type (crypt cells, villus cells or interspersed cells) from the same animal (Supplementary Table S1). A statistical analysis of these data shows that each cell type from each animal is statistically significantly different from all other cells in the same animal and from the cells of the same type in other animals, regardless of treatment (Supplementary Table S2). This suggests that the answer to questions 2, 3 and 4 from Figure 7 is that there are no similarities and that each subcellular portion of each cell is, statistically speaking, unique. And yet, when mean pixel values for each element are plotted for each cell type, subcellular compartment and animal, certain trends suggesting similarities became notable. For example, because of the high phosphorus content in DNA, in each and every cell type, P was always higher in the nuclei than in the cytoplasmic ROIs. This is visualized in Figure 8.

**Figure 9:**
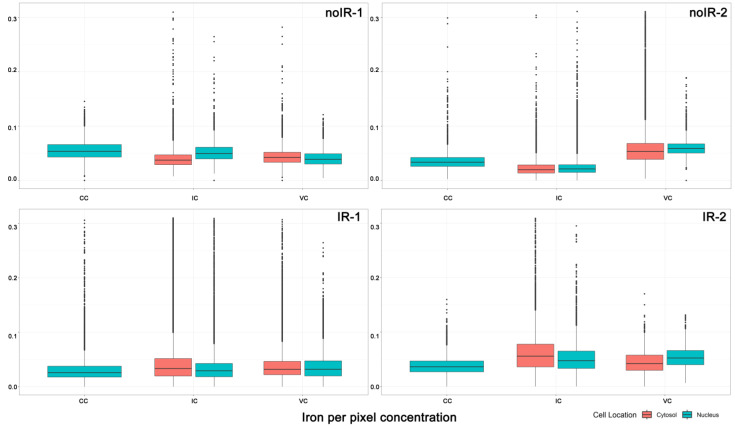
Individual pixel iron concentrations for all cell ROIs within a single animal: nuclei = teal and cytosol = pink. Three groups of data represent nuclei from crypt cells, cytosol and nuclei from interspersed cells and cytosol and nuclei from villus cells. For ROI generation, see Figure 4, Figure 5 and Figure 6. For corresponding numerical values, see Supplementary Table S1.

An equivalent bar graph showing the mean concentration of iron in different subcellular compartments is shown in Figure 9. The iron concentration of individual pixels is more heterogeneous than concentrations of other elements, with a greater presence of pixels with very high Fe concentrations. These pixels are the Fe hotspots that are especially visible in Fe XFM maps from irradiated tissues (Figure 2 and Figure 3 and Supplementary Figure S2). Associating these Fe-high pixels with cell or tissue features and evaluating the differences between tissues from sham irradiated vs. irradiated animals was one of the intentions of this work. Figure 9 shows the individual pixel concentrations for subcellular regions of specific cell types within an animal and demonstrates that Fe concentrations show change by subcellular compartment and by animal both. Some of the interspersed cells are likely macrophages, which are expected to have a high iron concentration in their cytosol after irradiation. Therefore, the observed higher mean Fe concentration in the cytosol (pink) compared to the nucleus (teal) of interspersed cells in irradiated animals aligns with our expectations.

#### 2.2.2. Mean Pixel Concentration Analysis—Individual Cell Analyses

**Figure 10:**
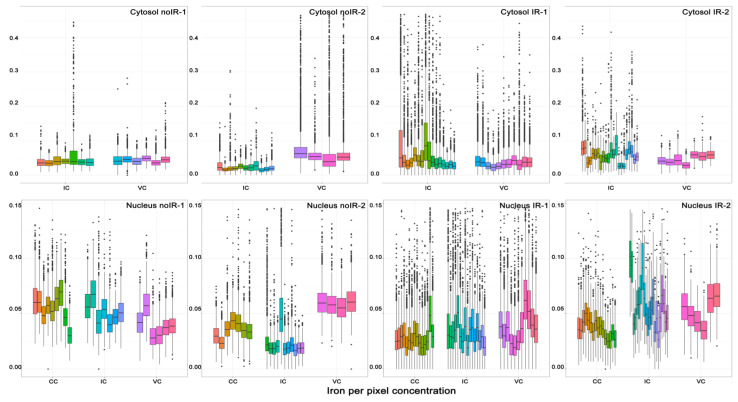
Individual pixel iron concentrations for separate subcellular compartments (cytosol = top, nuclei = bottom) for individual cell ROIs within each animal. For ROI generation, see Figure 4, Figure 5 and Figure 6. Note that *y*-axis maximum for cytosol is 0.4 micrograms per centimeter square, while it is only 0.15 for nuclei. Another issue worth noting is that majority of data for IR-2 animal comes from undersampled scans—scans generated with ~100 nm focal X-ray spot and 250 nm steps. Thus, IR-2 animal data show lower elemental concentrations than what is actually present in the sample (for more detailed explanations, see Section 1 and Section 4). Each cellular sub-compartment from each animal is indicated by a unique color.

In order to evaluate differences between individual cells, we also plotted mean iron concentrations for individual subcellular compartments of individual cells of different types (cells from different tissue regions) from different animals (Figure 10). This figure is congruent with Figure 9—cytosolic ROI from interspersed cells from irradiated mice have higher median Fe and/or a greater number of high concentration Fe pixels than interspersed cell cytosols from sham irradiated animals. At the same time, nuclei of interspersed cells did not show such differences. This result is highly encouraging as it appears that the increase in iron concentration found in intestines of irradiated animals can be mapped to the interspersed cells, many of which are expected to be macrophages. A more extensive screening of archival intestine samples from animals irradiated with external beam or exposed to internal emitters could permit us to establish a more nuanced understanding of the role of iron in complications caused by radiation exposure. Equivalent elemental concentration box plots for P, S, Ca, Cu and Zn are shown in Supplementary Figures S7–S11.

#### 2.2.3. ANOVA Evaluation of Cell-to-Cell Elemental Differences

Considering that the secondary goal of this work was to establish elemental cell classifiers that could be used for automated cell type recognition in XFM images, we compared whether subcellular regions of a specific cell type show significant element concentration differences from one another within the same cell type and for the same animal. For each element, each subcellular compartment of each cell type within an animal showed statistically significant differences based on an ANOVA test (Supplementary Table S2). Individual subcellular compartments of cells of the same type also show significant differences from each other even when specific elements are ignored (Supplementary Table S3).

This failure to discover elemental concentration similarities between subcellular compartments in cells of the same type inspired us to conduct comparisons between same cells scanned at two different sampling densities. In other words, we decided to pose question 1 described in Figure 7 (Will the statistical analysis recognize same subcellular regions from the same cell from two different scans as same?) in order to see whether our approach is biased to recognize individual cell differences, which then prevents us from discovering similarities between analogous cell types and cell regions.

#### 2.2.4. *T*-Test Evaluation of Differences between Different Scans of the Same Cells

The single-cell subcellular region ROI analyses of same cells scanned at two different sampling densities generated datasets that were different from each other. Six ROIs of crypt cell nuclei and ten more ROIs—five from nuclei and five from cytosols of five interspersed cells—were scanned with 100 or 250 nm steps using a ~100 nm beam (see Figure 3, Figure 4 and Figure 5). Mean per pixel elemental concentrations were obtained from regular and undersampling scans of the same subcellular regions of same cells. These values and their standard deviations are provided in Supplementary Table S4. Similar to ANOVA tests of the same cell types, *t*-test *p*-values for comparisons of the same subcellular regions from identical cells show many significant differences (Supplementary Tables S5 and S6). In order to segregate differences between identical subcellular areas of identical cells, we also calculated absolute percent differences of elemental per pixel concentrations for comparisons between regular and undersampled scans (Supplementary Table S7). Only when elemental concentration differences greater than 5% were used as a cutoff to apply *p*-values from *t*-test, some of the same subcellular regions of the same cells scanned at different step sizes showed themselves to be the same (Supplementary Table S8).

#### 2.2.5. Tissue Region Analyses

Considering that we were unable to generate elemental classifiers for individual cells, we decided to work with larger tissue regions such as crypt regions and interspersed cell regions. ROI analyses (Supplementary Figures S3–S6) were used to generate elemental concentration data for individual pixels from the entire tissue regions. Mean per pixel elemental concentrations for the entire tissue regions were calculated (Supplementary Table S9). Both types of tissue regions included many cells. Crypt cell regions were used for analysis only when they clearly represented crypt morphology. Interspersed cells regions constitute the entire area underneath the villi surface and excluded crypt regions. Thus, while crypt regions included crypt stem cells and Paneth cells, the interspersed regions included “interspersed cells”, many of which are macrophages that were labeled with the F4/80 antibody as shown in Figure 1 and Figure 2 and Supplementary Figures S1 and S2. While mean per pixel concentrations of iron were greater in crypt regions than in interspersed regions of sham irradiated, the opposite was true for the irradiated mice. There, the interspersed cell regions had higher or equal mean iron concentration compared to crypt tissue regions. An Anderson–Darling test conducted with these data showed statistically significant differences between crypt regions and interspersed cell regions for all elements (Supplementary Table S10).

**Figure 11:**
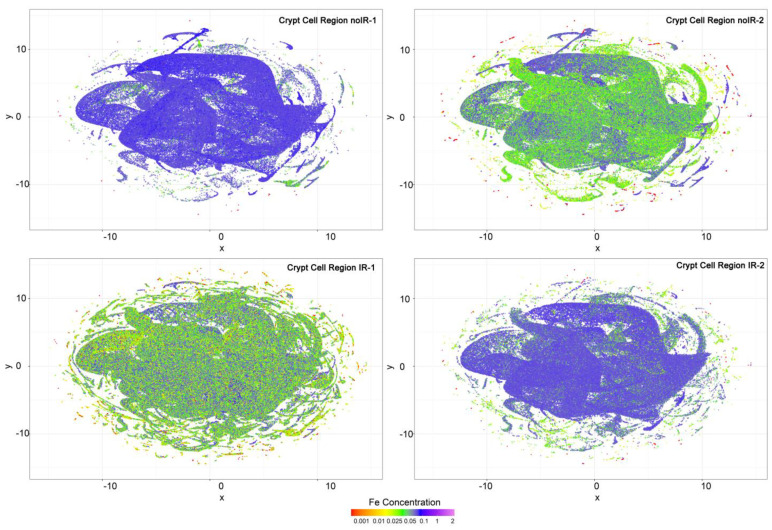
UMAP graphs generated for all six elements for crypt cell regions were colored to display iron concentrations as shown in the color bar. The four graphs show modest differences in iron content but no distinctive association between Fe per pixel concentration and a specific UMAP data pattern that correlates to radiation exposure. Elemental concentration scale bar shows colors corresponding to different concentrations of iron expressed in micrograms per centimeter square.

**Figure 12:**
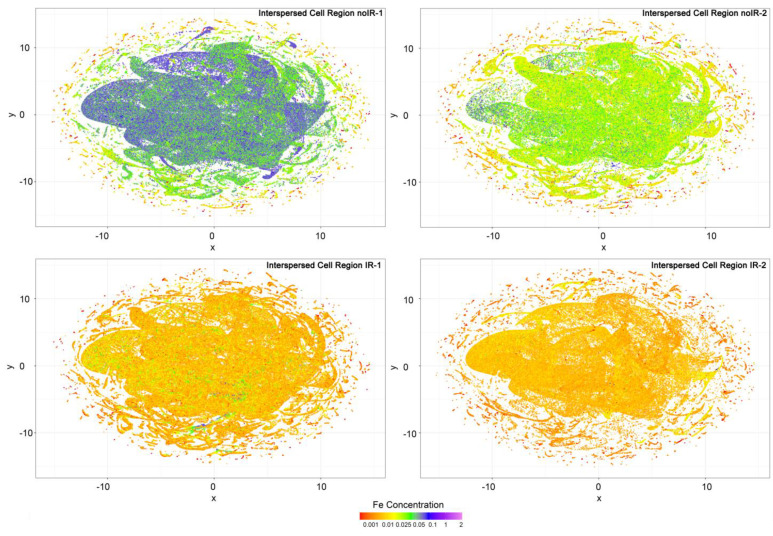
UMAP graphs generated for all six elements for interspersed cell regions were colored to display iron concentrations as shown in the color bar. These four graphs show differences in iron content as well as distinctive association between Fe per pixel concentration and a specific UMAP data pattern. Elemental concentration scale bar shows colors corresponding to different concentrations of iron expressed in micrograms per centimeter square.

In our effort to develop elemental classifiers that would depend on multiple elements at the same time, we conducted UMAP analyses because they reduce the data to two dimensions that can be plotted by animal and tissue region. When both crypt and interspersed cell regions from all four mice were compared with each other, no marked differences in UMAP distribution were notable (Supplementary Figure S12). Similarly, when all crypt regions (Supplementary Figure S13) or interspersed cell regions (Supplementary Figure S14) were combined and the mice compared with each other, no obvious differences in data distribution were seen. However, while each *x*, *y* position in a UMAP graph depends on all six elements included in the elemental concentrations ROI analysis (P, S, Ca, Fe, Cu and Zn), these datapoints can be colored to represent concentrations of only a single element. Because iron is the element in which we are interested the most, the UMAP graphs were colored to show the concentration of iron in addition to showing data distribution for six elements for crypt tissue regions (Figure 11) and interspersed cell regions (Figure 12).

Interestingly, in all animals, datapoints with the lowest Fe concentration are in the outermost regions of the UMAP data maps. Also, surprisingly, a casual visual inspection of Figure 12 suggests that the overall concentrations of Fe are higher in interspersed cell regions in non-irradiated mice. At the same time, we know that in sham irradiated mice, mean per pixel Fe concentrations are 0.04 and 0.02 micrograms per centimeter square, while they are 0.06 and 0.04 in irradiated animals. This suggests that the datapoints that are responsible for majority of the Fe signal in irradiated animals are relatively few and because of that, do not feature prominently in UMAP images. The relevance of this unevenness of Fe distribution may be at the root of ferroptosis in irradiated intestine samples. However, additional work will need to be undertaken to confirm or refute this notion.

## 3. Discussion

GI-ARS is a fatal outcome of exposure to high doses of whole-body irradiation and currently with no treatment options despite decades of research into possible countermeasures [28]. Considering that one of the features of GI-ARS is an increase in iron concentration in the irradiated intestine, rapid and reliable imaging of iron in all of its chemical and biological forms would be a tool that could be used to engage with GI-ARS and to explore modalities that show potential to mitigate it. A technique that could fill this need is XFM, and while this technique cannot be used with living organisms, it could possibly be used on cells sloughing off from the inner surface of the intestine. However, while rapid approaches for the collection of such cells would probably be easy to develop, rapid XFM analysis is still beyond our reach. In addition to developing instruments for routine XFM imaging, independent of synchrotron access, data analysis itself needs to have much higher throughput than it is now. It would be extremely useful to develop a pipeline for the automated elemental evaluation of numerous tissue samples and revolutionize the generation of ROIs for tissue regions, single cells and subcellular regions. We propose that this potential new software could be based on elemental content combinations. Our attempts to generate such a tool are still far from meeting this need. Nevertheless, this work presents an early attempt in this direction.

Research using X-ray fluorescence to analyze geological samples has yielded approaches for bulk sample principal component analyses that can be used to segregate between different obsidian samples, such as e.g., [29]. Equivalent efforts to generate elemental classifiers for other samples including intestinal samples are lacking so far. New approaches for the elemental quantification of XFM data of biological samples are needed—especially such approaches that would aid with recognizing and handling sample heterogeneities. One avenue to reach this goal is to use complementary imaging techniques in combination with XFM. Three recent examples include the following: the use of Fourier transform Infrared Spectroscopy [30], which was recently used specifically to cross-correlate XFM data against sample thickness and density [31]; the use of electron microscopy, where a recent example shows elemental quantification of subcellular organelles coupled with organelle identification [32]; and the use of low-dose in-line holography, which generates electron density maps used subsequently to guide XFM at nanofocusing beamlines [33]. However, while powerful, correlative imaging often comes with significant operational costs, the development of sample substrates and sample holders that work for different imaging techniques is still in its early stages and sample holders and imaging environment are very different in different instruments as well. The situation is slightly less complicated when other X-ray imaging techniques are used, such as, for example, ptychography [34]—a powerful X-ray contrast-based technique, which was recently used to image metaphase chromosomes [35]. Nevertheless, even if a sample can be studied with two imaging modalities with relative ease, it is difficult to imagine a situation where a researcher could routinely use multiple techniques to collect high volumes of data. Thus, while different types of correlative imaging should be used to verify XFM findings, generating robust stand-alone high throughput XFM imaging approaches is necessary in order to advance XFM to an OMICs compatible level. This goal for XFM development is the only one that is compatible with the use of XFM for sample screening that would satisfy the demands of diagnostics, pathology and other areas of biomedical research focusing on complex questions.

XFM has been used to determine the elemental content of different subcellular organelles in single cells [32,36]. Several attempts to develop automated approaches for identification of subcellular organelles were made for erythrocytes [37], cells in culture [38] and bacteria [39]. However, the complexity of tissue samples is such that it is difficult to develop equivalent approaches that would be able to classify different tissue regions and different cells and cell subregions without acquiring elemental data at high densities. In this work, we have tried several different approaches to reach this goal, but we encountered limitations that are suggestive of a future success requiring additional work. Differences in data density between different scans were found to prevent regular statistical analyses from uncovering sample identity (Figure 3, Supplementary Tables S4–S8). The inspection of these images confirmed that sampling differences make it difficult to make sample-to-sample comparisons. At the same time, the acquisition of XFM data at the same density is sometimes not possible due to limited access to instruments. Therefore, it will be necessary to develop flexible approaches to equalize data density between scans. This will require that the pixel information include not only elemental information but also information about pixel size, motor positions at the time of data acquisition, etc. Moreover, this type of detailed pixel data will help with the recognition of cell features that can be expected to spread over several contiguous pixels, etc. Approaches to generate this new type of data output are in progress. Considering that our team was able to master reconstructions of tomographic XFM data [20,25,40], we are hopeful that the development of elemental classifiers will eventually generate an approach for automatic and high throughput ROI analysis of XFM data. In addition, we found that using UMAP for dimensionality reduction (Figure 11 and Figure 12) may be a promising avenue to reach this goal.

## 4. Materials and Methods

Samples. A previous study focused on GI-ARS [3] generated the samples that we used for XFM imaging in this work. Briefly, small intestines were obtained from two pairs of BALB/c mice, a pair of sham irradiated controls, and a pair of animals exposed to 8 Gy of cesium 137 Cs gamma rays at a dose rate of 95.7 cGy/min (Best Theratronics, Ottawa, ON, Canada). Irradiations occurred one week before animal sacrifice and organ harvest. This mouse strain had a reported LD50/30 of 5.7 Gy [41] and exposure to 8 Gy dose caused GI-ARS as shown by changes in crypt and villi morphology at 7 days after exposure [3]. Formalin fixed tissue was paraffin embedded and sectioned at a thickness of 5 micron for all imaging analyses. In the earlier study, the samples were stained with hematoxylin and eosin (H&E) or used for immunohistochemistry for proteins including Cyclin D1, COX-2, and macrophage marker F4/80 [3]. Visible light images of samples labeled for F4/80 at the time of that work were included in this work (Figure 1 and Figure 2 and Supplementary Figures S1 and S2).

XFM scanning. For this work, newly sectioned 5 micron thick small intestine sections were mounted on 200 nm thick silica nitride (Si_3_N_4_) windows (Silson, Southam, UK) with 1 × 1 mm window area. XFM imaging was performed at Sector 9 of the APS with the Bionanoprobe instrument [18,19,20]. A beam spot size of ~100 nm FWHM was generated by Fresnel zone plate focusing, and the samples were imaged at room temperature using different step sizes.

MAPS analyses. Raw X-ray fluorescence spectra were calibrated using AXO thin sample standards and a standard normalization procedure as we have undertaken in the past [20,21,23,26,27]. For each sample, numerous regions of interest (ROIs) were generated, focusing on different areas of the sample as detailed in the Results section. For the purpose of tissue region analyses, ROIs that were generated either corresponded to entire crypts or areas with interspersed cells. The latter corresponded to regions between surface of villi, excluding the surface cell layer, and the outer perimeter of the intestine excluding the areas with crypts. In cell type analyses, ROIs were drawn to correspond to single-cell nuclei or single-cell cytosol regions. Please note that without immediately adjacent tissue sections stained by immunohistochemistry it is not possible to determine specific cell types. Thus, while many of the interspersed cells may be macrophages, their exact identification would require that a portion of the same cell be present in an adjacent slice of the tissue and identified using IHC. Similarly, different cell types are present within the crypts and villi as well. For the purpose of this work, we decided to simply talk about cells within a tissue region as their only identifying designation. Hence, we split the cells into only three general categories: crypt cells were all cells within clearly outlined crypt regions; villus cells were cells comprising the villus surface; interspersed cells were all cells that could not be associated with either of the two other structures (crypts and villus surface) but were located between them. The scatter plot data function in MAPS software was used to extract elemental concentrations determined for each pixel within the ROIs investigated. Per pixel information including ROI origin and concentrations for phosphorus (P), sulfur (S), calcium (Ca), iron (Fe), copper (Cu) and zinc (Zn) in micrograms per centimeter square were used for statistical analyses.

### Statistical Analyses

Variables/Data Summaries. Three sets of data were configured from the collected pixel-by-pixel element content. Each dataset contained a row per pixel of the scan, with element concentrations of P, S, Ca, Fe, Cu and Zn per row and identifiers that indicated the mouse, cell location within the tissue, and/or tissue regions important to the necessary analysis. The elemental concentration distribution was visualized using boxplots by element and mouse grouped by cell type. Element concentration per pixel was also summarized by element, mouse, tissue region, cell type and cell sub-region using mean and standard deviations.

ROI Data Analysis. The ROI data for subcellular regions of single cells had two regions, cytosol and nucleus, with three cell types: crypt cells (CC), villi cells (VC), and interspersed (IC) cells. Within each of these cell groupings, there were individual cells such as CC 1, CC 2, etc. that added a layer of analysis. After data visualization and summaries, mean per pixel elemental concentrations in different ROIs and groups of ROIs were calculated in R (Supplementary Tables S1 and S4), and ANOVAs were conducted for each combination of mouse and cell type to identify if there were differences between individual cells for each element concentration. To account for multiple testing, all p-values were corrected using the false discovery rate (FDR) adjustment (Supplementary Tables S2 and S3). Uniform Manifold Approximation and Projection (UMAP) was conducted to reduce the data for six elemental concentrations to two dimensions that could be plotted and grouped by mouse, tissue region, or cell type as needed.

Multiple Scans of Same Cell Analysis. The scan data only contained scans from one mouse with two scans for the same tissue, with similar layers of cell regions, group and type as above. The goal of this analysis was to determine a method to accurately test similarities between cell types and potentially overcome the issues of statistical analyses of high-density data. To compare the element concentrations in identical subcellular regions of identical cells between regular scans (where X-ray beam and step size match) and undersampled scans (where step size was 2.5 × greater than the X-ray beam), a t-test was conducted with FDR adjustment to account for multiple testing. Additionally, an absolute 5% or more difference in median concentration criterion was applied to refine the accuracy of distinguishing the cell groupings (Supplementary Tables S6–S8). Using such a criterion can filter out minor changes that, while statistically significant, may not be biologically or clinically relevant [42].

Tissue Region Analysis. Tissue region data consisted of either crypt region or interspersed cell regions in different mice per each element, with no additional layer of groupings. Once again, mean per pixel elemental concentrations were calculated in R (Supplementary Table S9). An Anderson–Darling test was also conducted to identify differences of elemental concentration between tissue regions for each animal (Supplementary Table S10). UMAP was used for dimensionality reduction, allowing for the visualization of variance.

All analyses were conducted using R version 4.3.1. The R code used for all analyses shown here is currently available on our research group GitHub webpage (https://github.com/WoloschakLab/GeneralCode (accessed on 16 September 2024)).

## 5. Conclusions

This study had two goals—to demonstrate that iron redistribution that occurs in intestinal samples of irradiated animals can be documented using XFM, and to attempt to uncover unique combinations of elemental concentrations that would aid in the development of classifiers that could be used to generate ROIs automatically. Our first objective was met—iron concentration was found to be higher in the irradiated intestine at 7 days after exposure, where iron was found in the intercellular area of the interspersed cell tissue region. Our second objective will require additional work, although our results are promising. We anticipate that the use of pixel location coordinates, accessible using new software that is in development as MAPS replacement, could provide guidance for pixel attribution into different cell area clusters and aid in this work.

## Data Availability

The R code used for all analyses shown here is currently available in our research group GitHub webpage (https://github.com/WoloschakLab/GeneralCode, accessed on 16 September 2024).

## Supplementary Materials

The following supporting information can be downloaded at: https://www.mdpi.com/article/10.3390/ijms251910256/s1.
