# Supplementary material for: X-ray Fluorescence Microscopy to Develop Elemental Classifiers and Investigate Elemental Signatures in BALB/c Mouse Intestine a Week after Exposure to 8 Gy of Gamma Rays
Source: Int J Mol Sci. 2024 Sep 24;25(19):10256. doi: 10.3390/ijms251910256 (PMC11477073; doi:10.3390/ijms251910256)
Supplement: Supplementary file 1 [file ijms-25-10256-s001.zip › Suplemental Figures.pdf]

X-ray Fluorescence Imaging to Develop Elemental Classifiers and Investigate Elemental Signatures in BALB/c Mouse Intestine a Week after Exposure to 8 Gy of Gamma Rays

Anthony Smith[1], Katrina Dobinda[1], Si Chen[2], Tatjana Paunesku[1], Zequn Sun[1,*] and Gayle Woloschak[1,*]

[1] Feinberg School of Medicine, Northwestern University

[2] X-ray Imaging Division, Advanced Photon Source, Argonne National Laboratory

Supplemental Figures

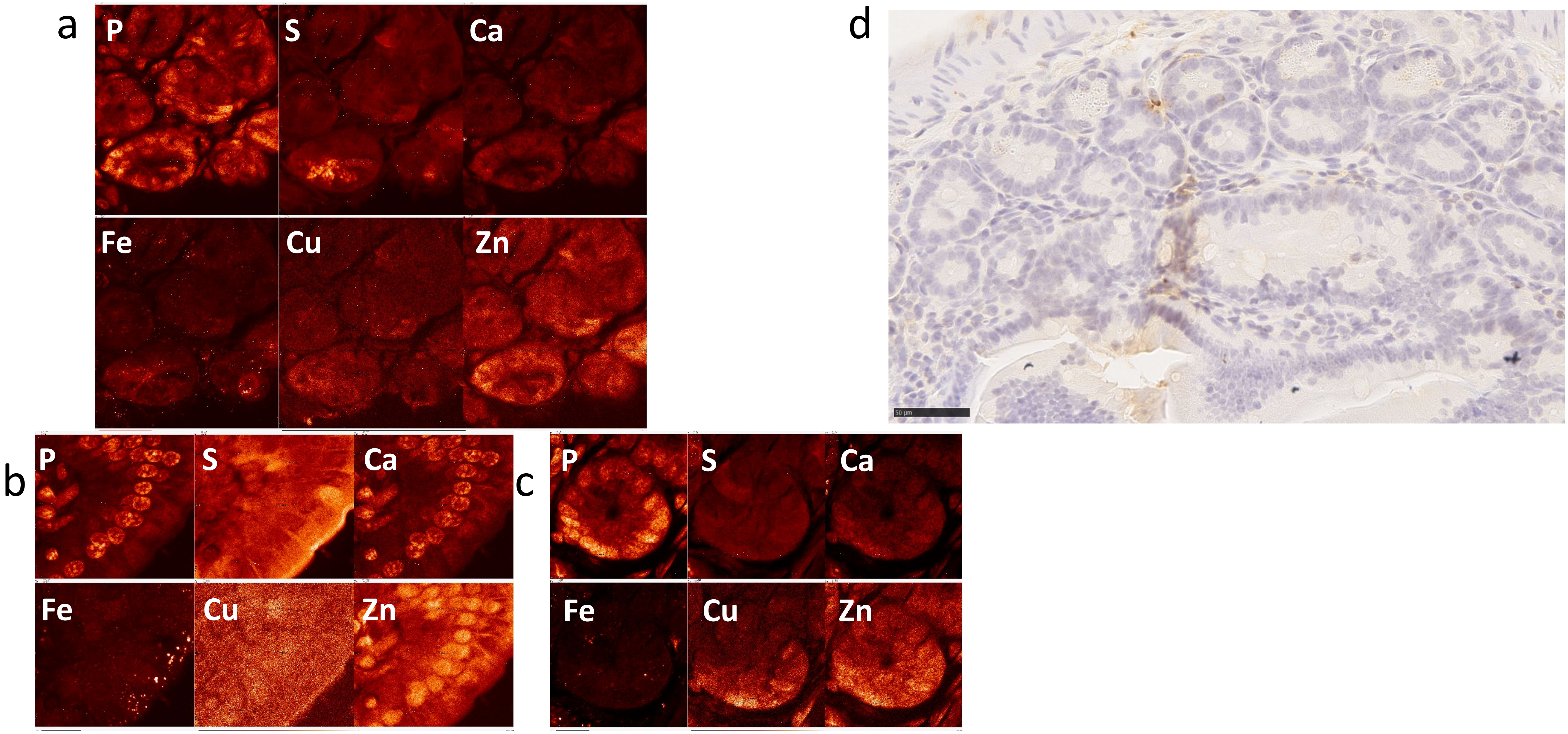

Supplemental Figure S1. Elemental maps of intestine of the sham irradiated mouse noIR-2 scanned with ~100 nm x-ray beam. Three different areas of the intestine are presented (a,b,c) and a visible light image of a similar area of the intestine – IHC for F4/80(d). Step sizes were 100 nm, and scan areas sizes 70 by 80, 41 by 43 and 36x40 micron, respectively. Scale bars in a, b, and c indicate 20, 10, and 10 microns, scale bar in (d) is 50 microns.

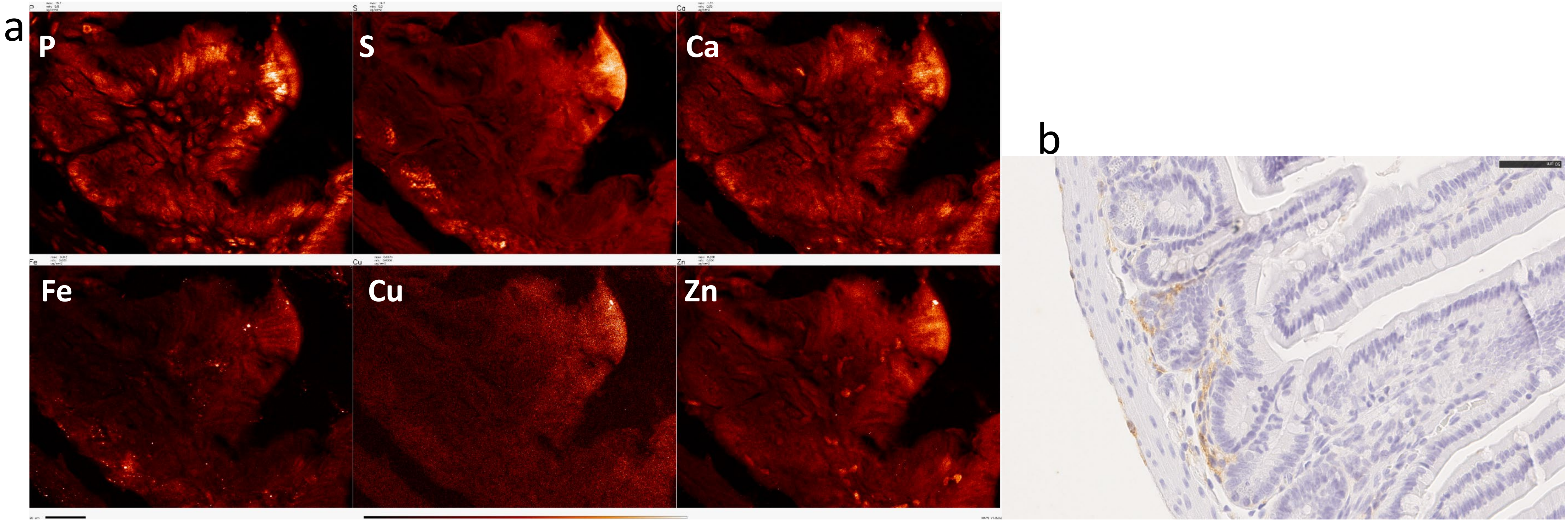

Supplemental Figure S2. Elemental map of intestine of the irradiated mouse IR-2 scanned with ~100 nm x-ray beam. Scan of an area of the intestine is presented (a) and a visible light image of a similar region – IHC for F4/80 (b). Scale bar in (a) is 20 microns, in (b) 50 microns.

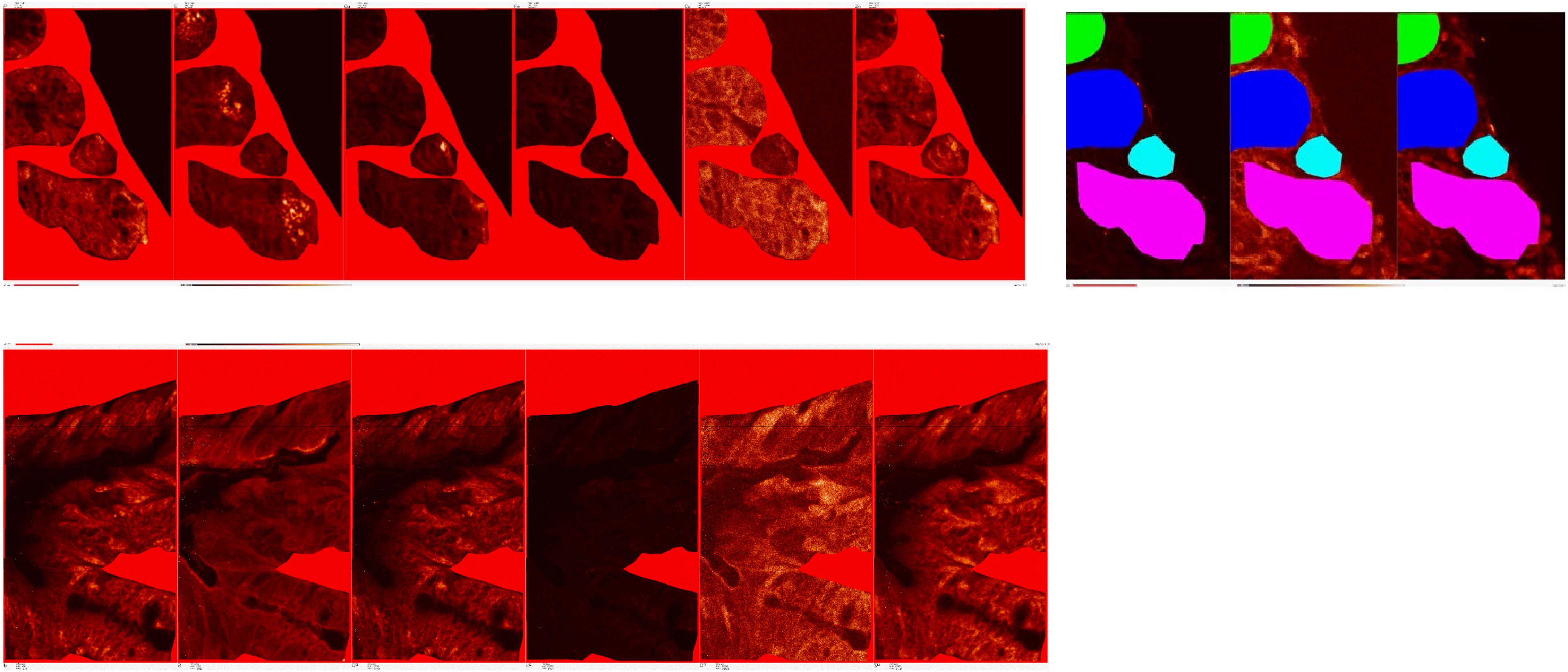

Supplemental Figure S3. Sham irradiated mouse no-IR-1: tissue region ROIs of the interspersed cell regions (red ROIs) and crypt regions (green, navy, turquoise, magenta).

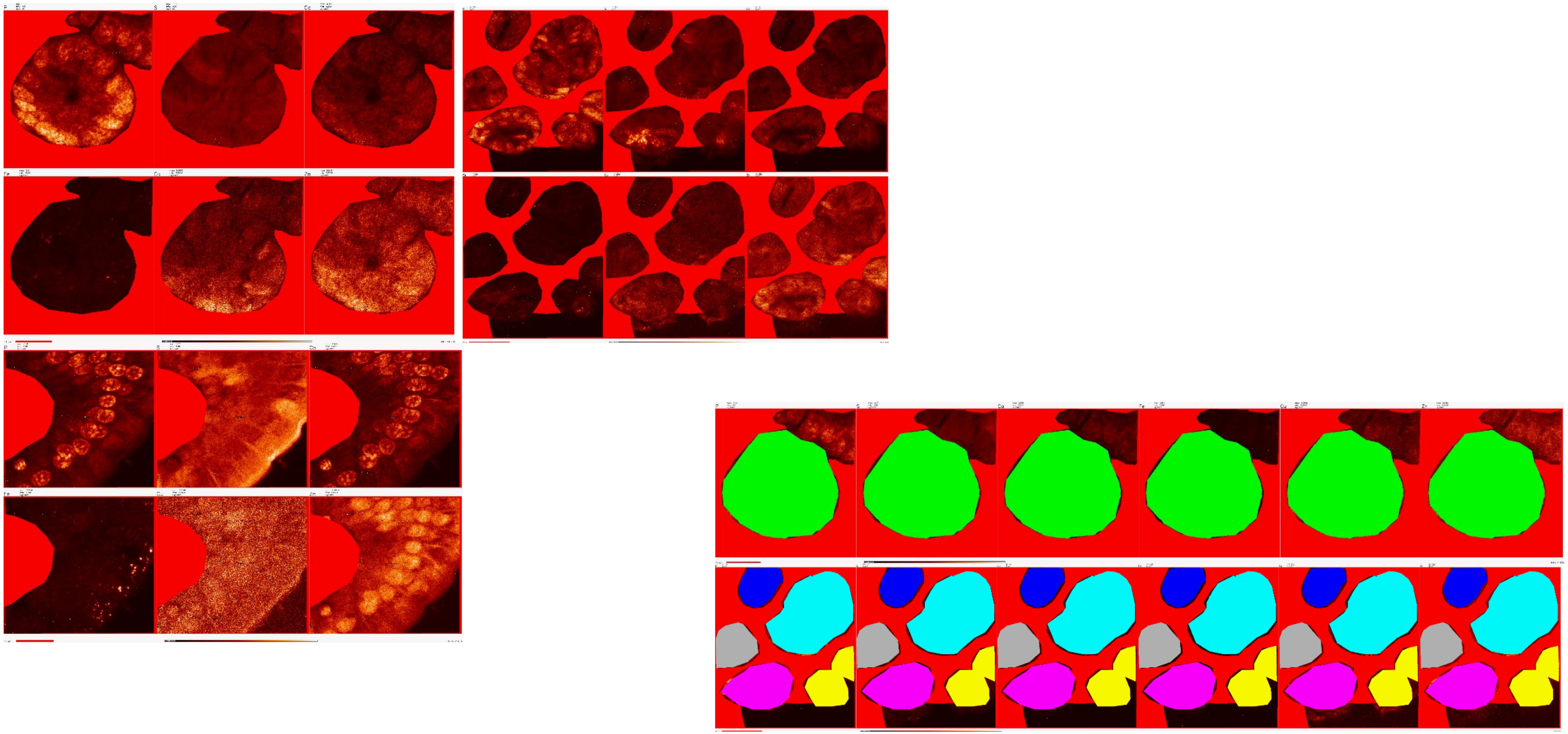

Supplemental Figure S4. Sham irradiated mouse noIR-2: tissue region ROIs for interspersed cell regions (red ROIs) and crypt regions (green, navy, turquoise, magenta, yellow, gray)

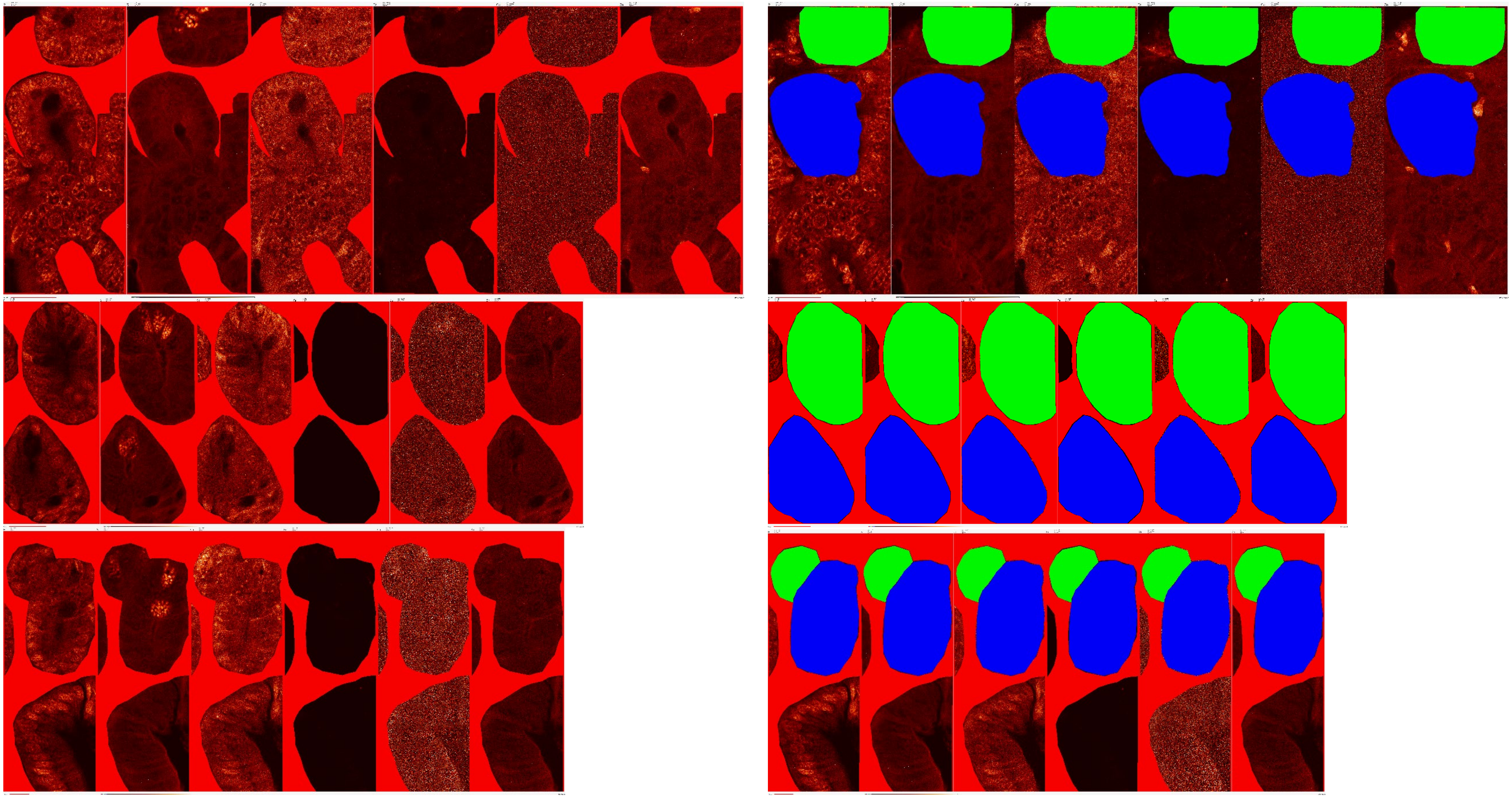

Supplemental Figure S5. Irradiated mouse IR-1: tissue region ROIs for interspersed cell regions (red ROIs) and crypt regions (green, navy).

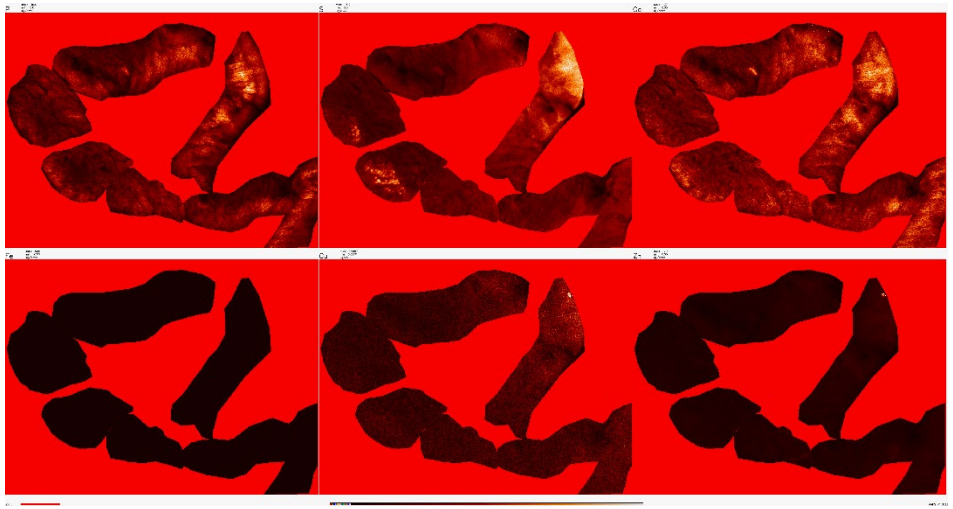

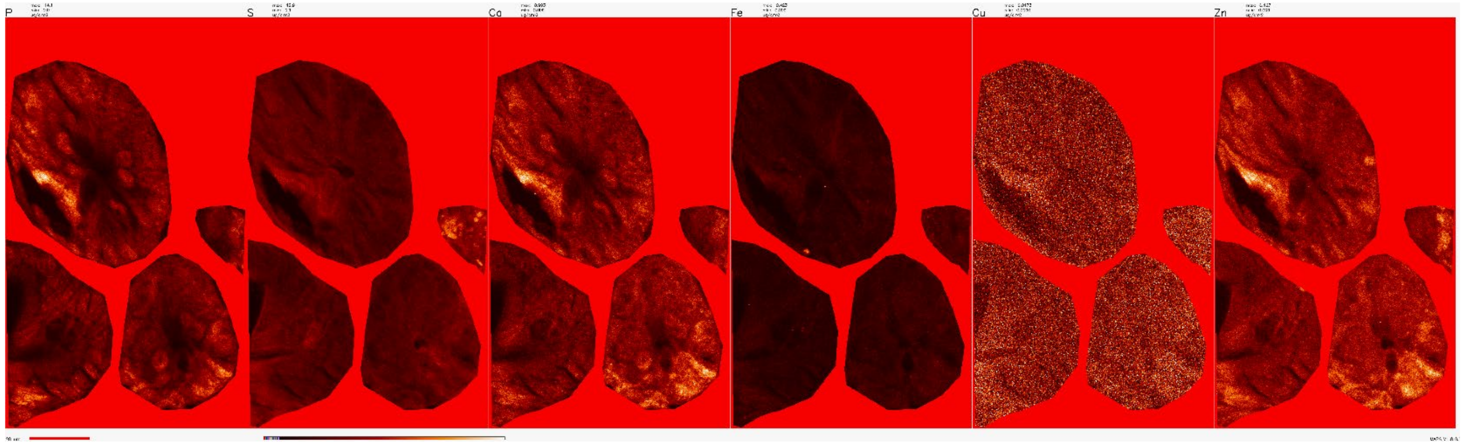

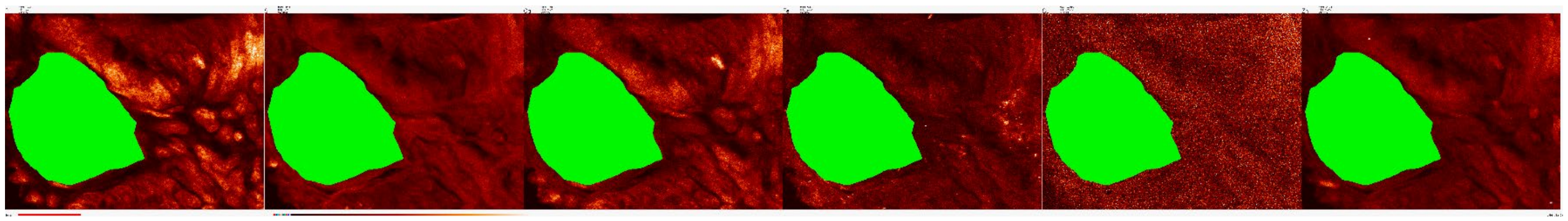

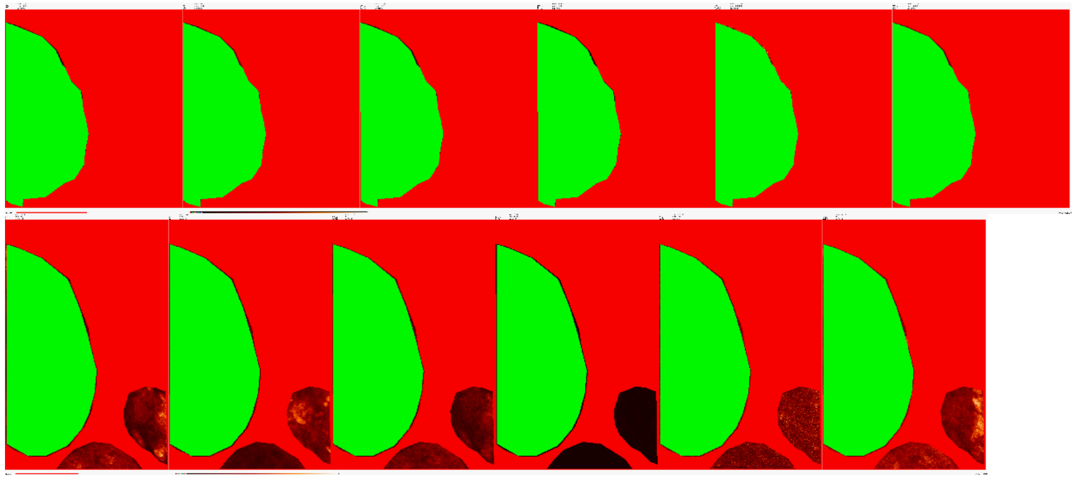

Supplemental Figure S6. Irradiated mouse IR-2: tissue region ROIs for interspersed cell regions (red) and crypt regions (green)

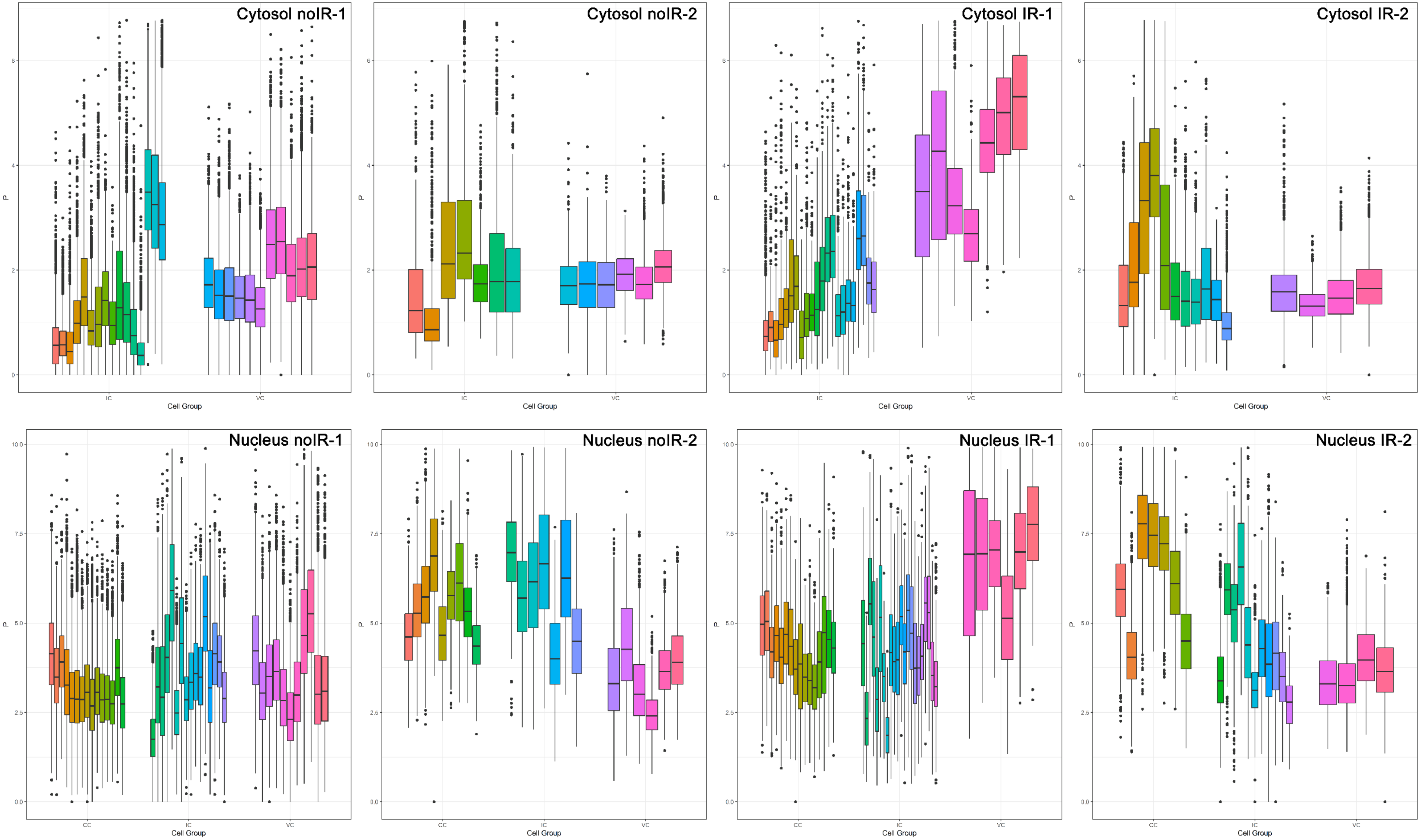

Supplemental Figure S7. Individual pixel phosphorus concentration for separate subcellular compartments (cytosol = top, nuclei bottom) for individual cell ROIs within each animal. For ROI generation see Figures 4-6.

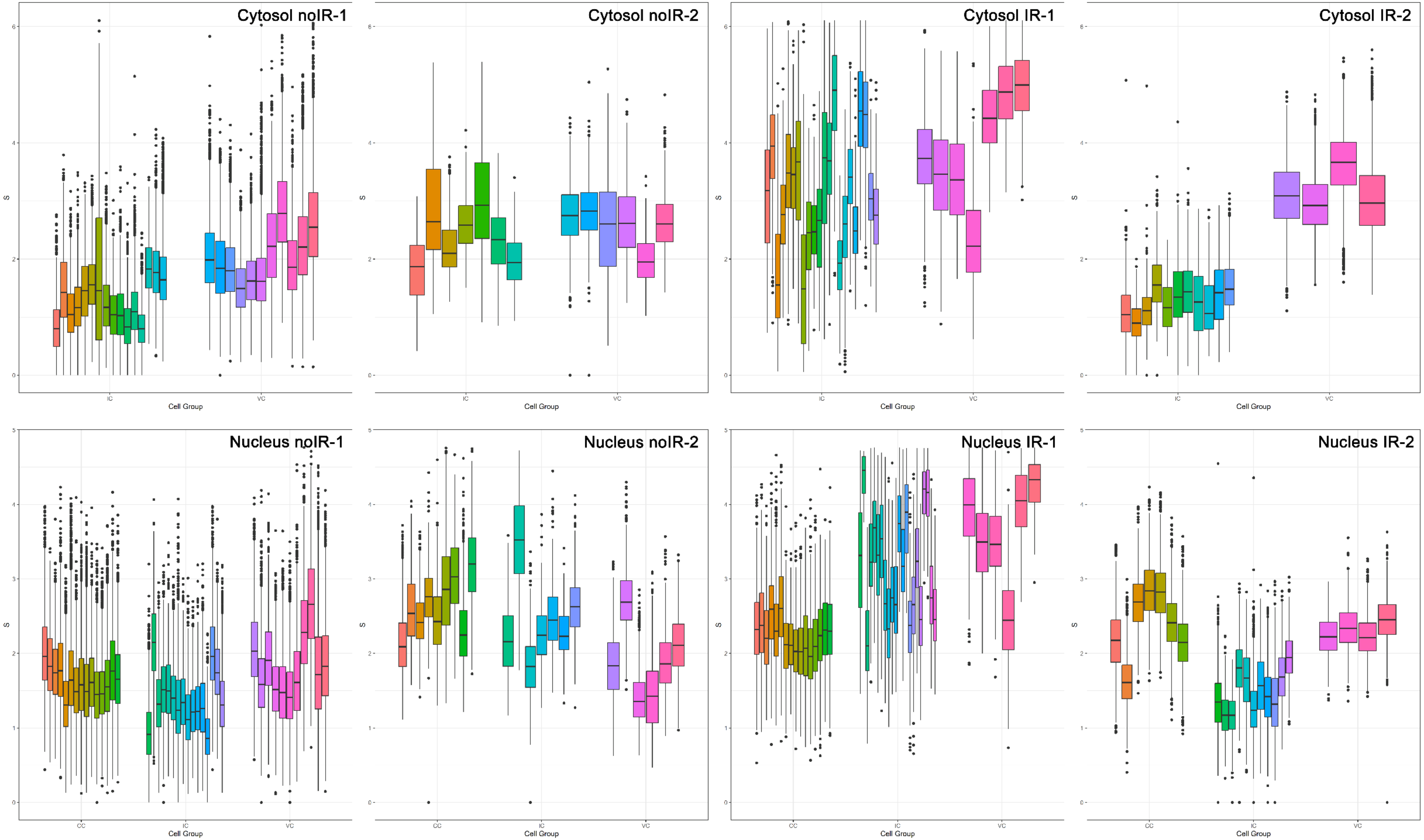

Supplemental Figure S8. Individual pixel sulfur concentration for separate subcellular compartments (cytosol = top, nuclei bottom) for individual cell ROIs within each animal. For ROI generation see Figures 4-6.

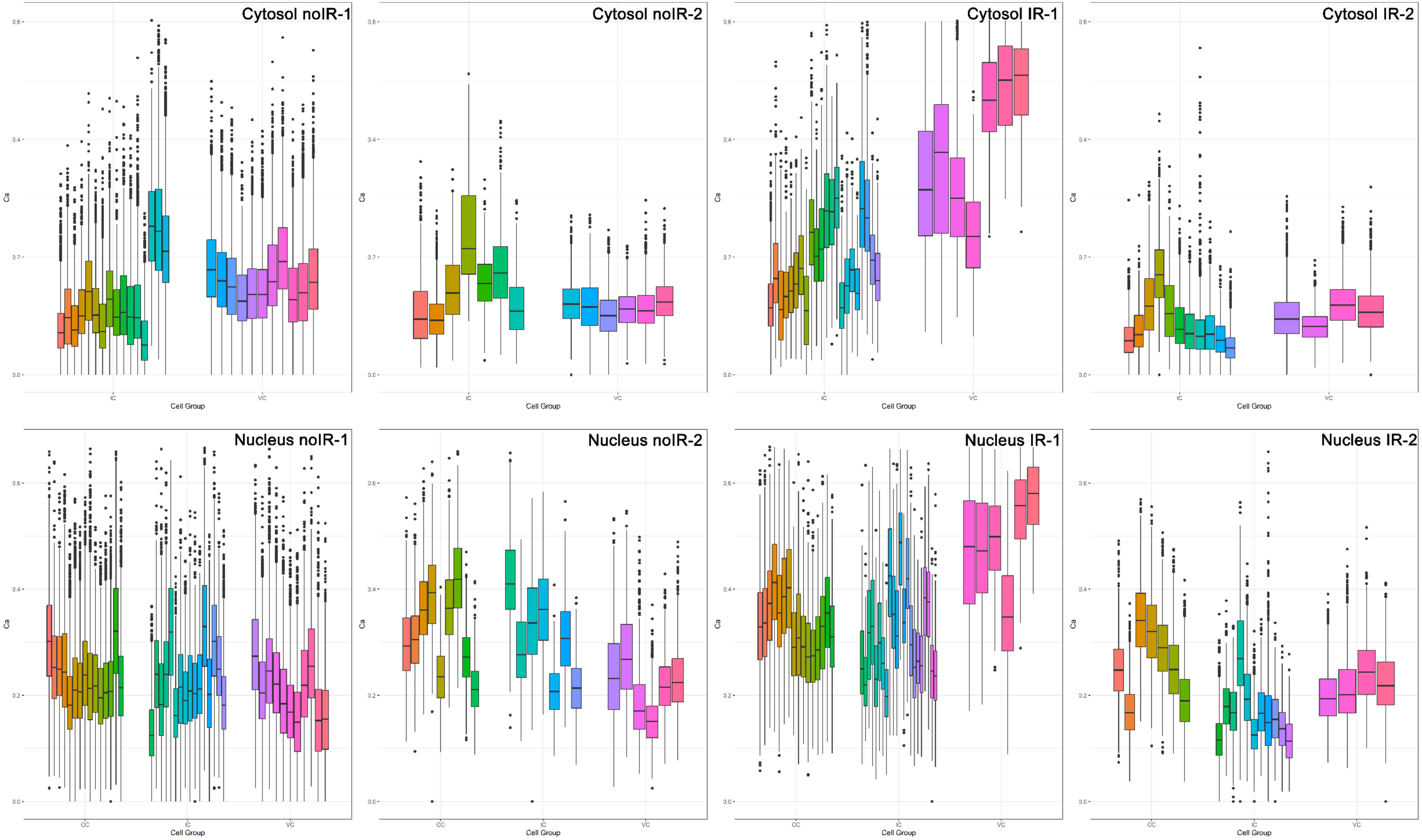

Supplemental Figure S9. Individual pixel calcium concentration for separate subcellular compartments (cytosol = top, nuclei bottom) for individual cell ROIs within each animal. For ROI generation see Figures 4-6.

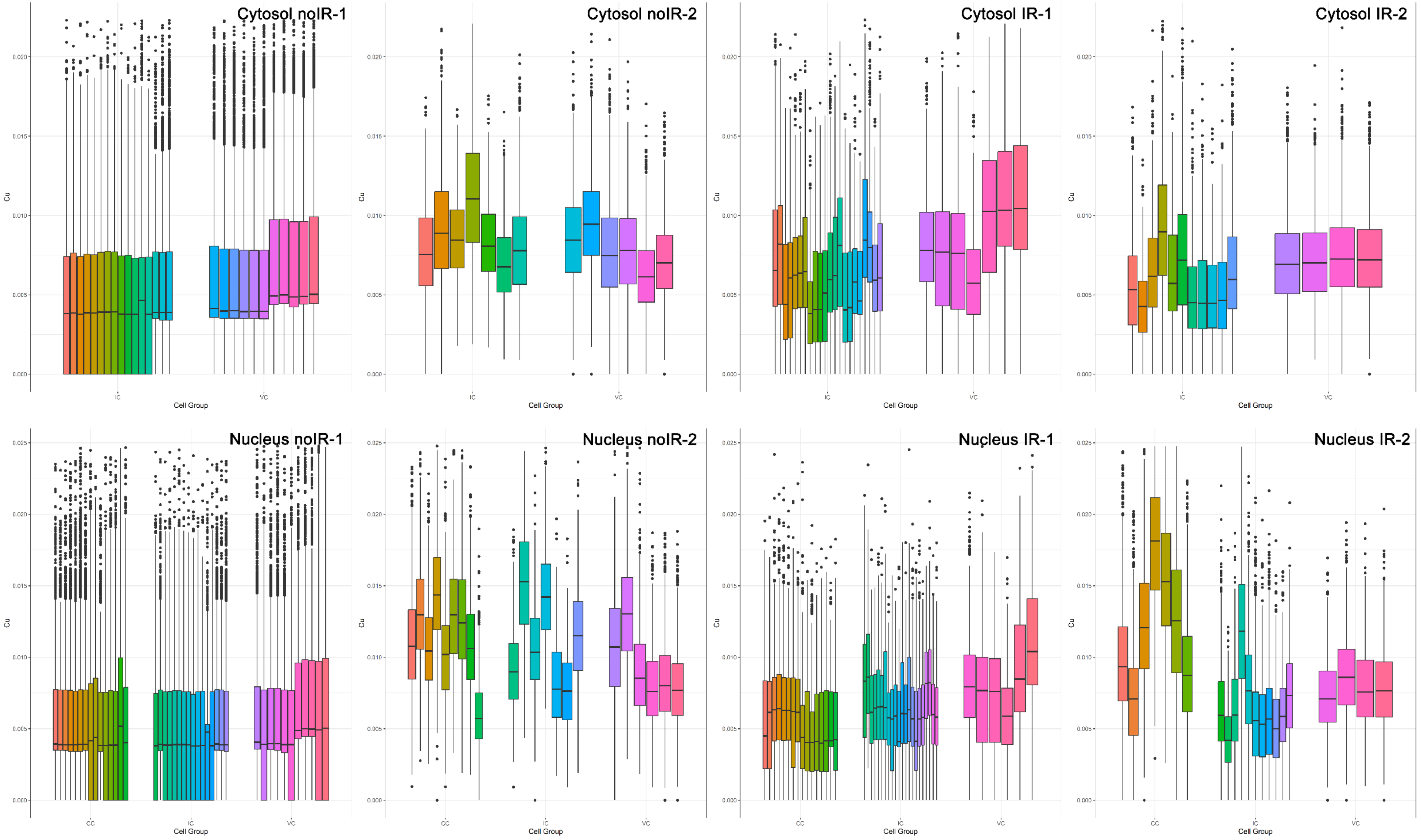

Supplemental Figure S10. Individual pixel copper concentration for separate subcellular compartments (cytosol = top, nuclei bottom) for individual cell ROIs within each animal. For ROI generation see Figures 4-6.

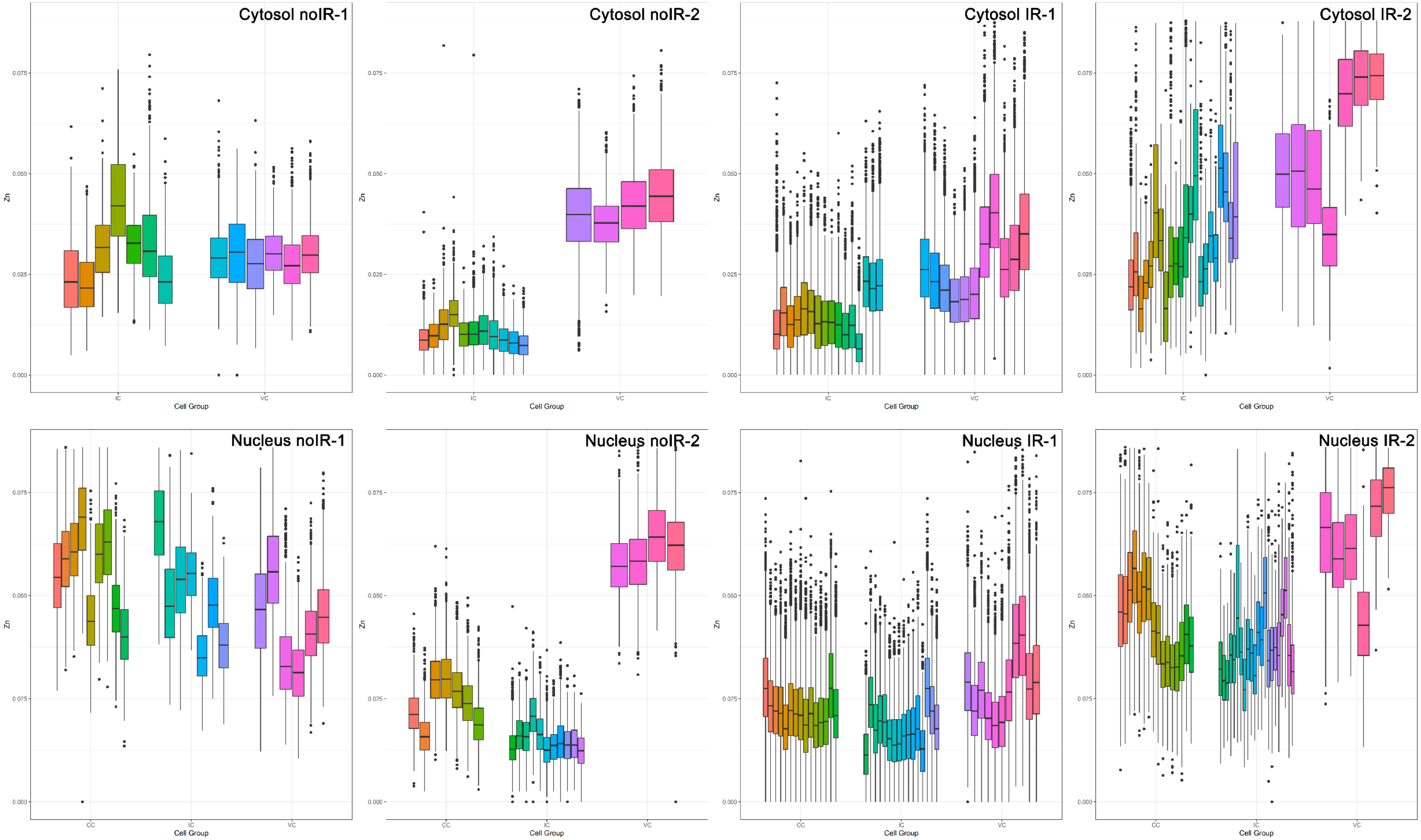

Supplemental Figure S11. Individual pixel zinc concentration for separate subcellular compartments (cytosol = top, nuclei bottom) for individual cell ROIs within each animal. For ROI generation see Figures 4-6.

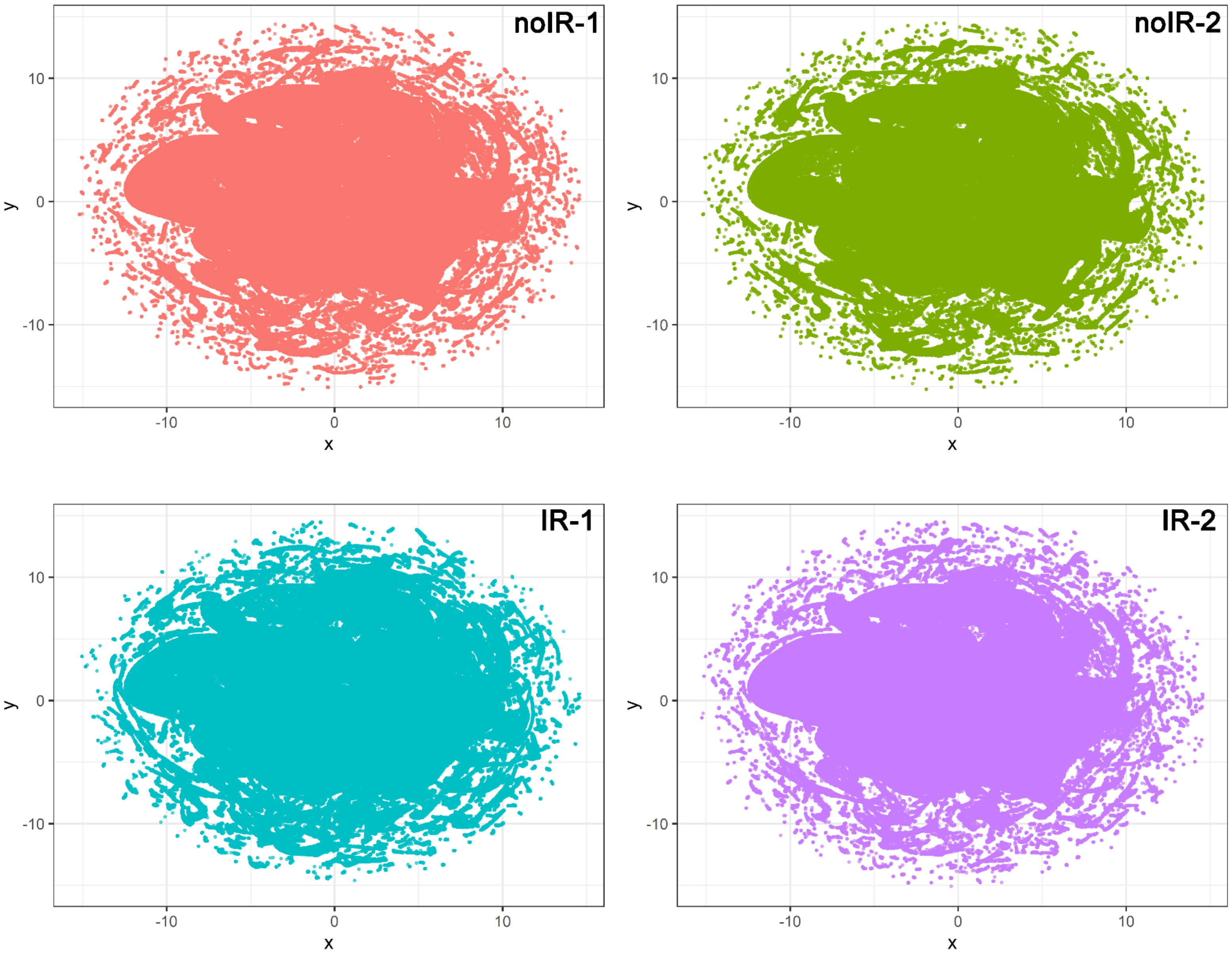

Supplemental Figure S12. UMAP analysis of all per pixel concentrations for all tissue regions from all animals. For ROI generation see Supplemental Figures 3-6.

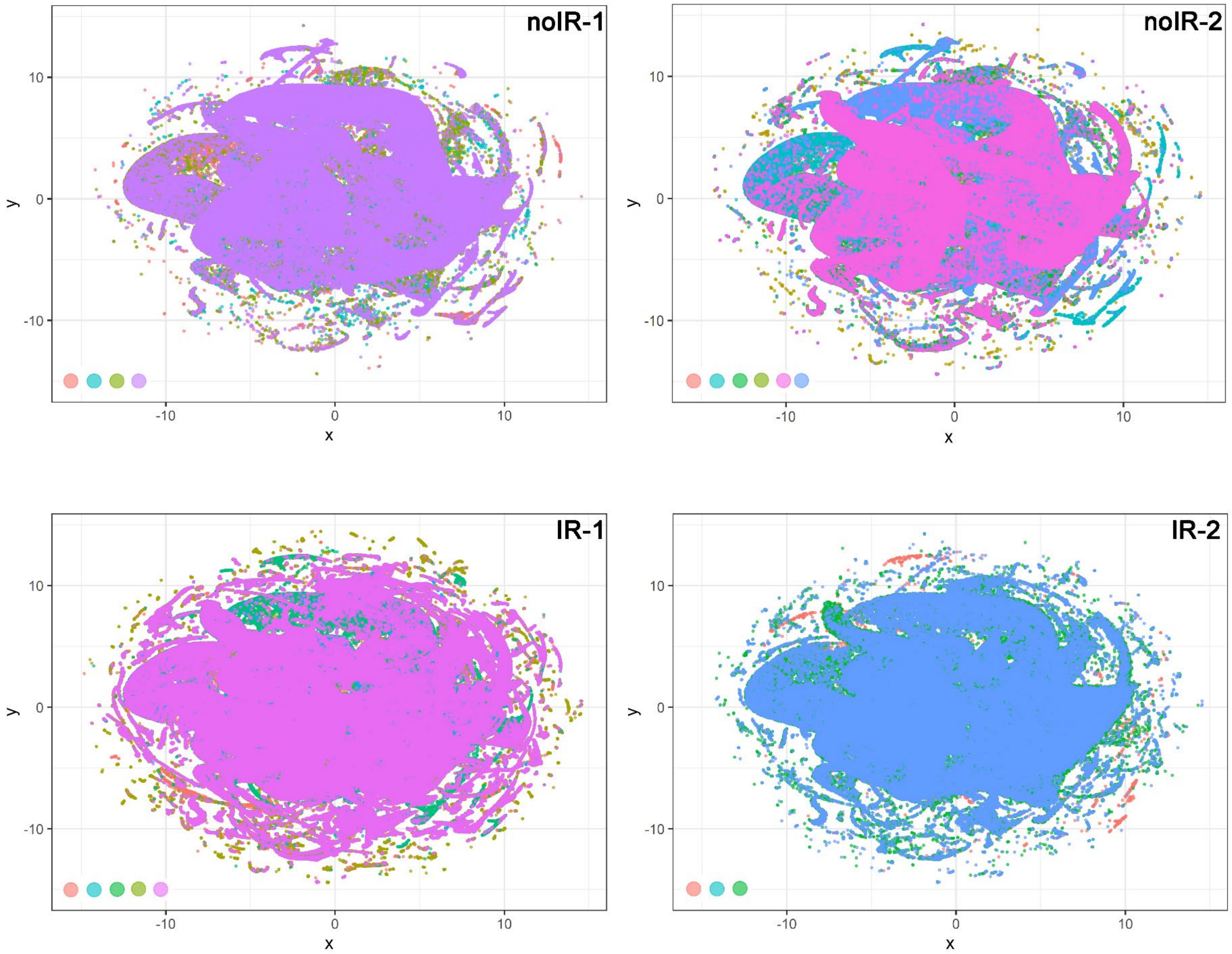

Supplemental Figure S13. UMAP analysis of all per pixel concentrations for all crypt tissue regions from all animals. For ROI generation see Supplemental Figures 3-6. Individual crypt regions are represented by different color dots.

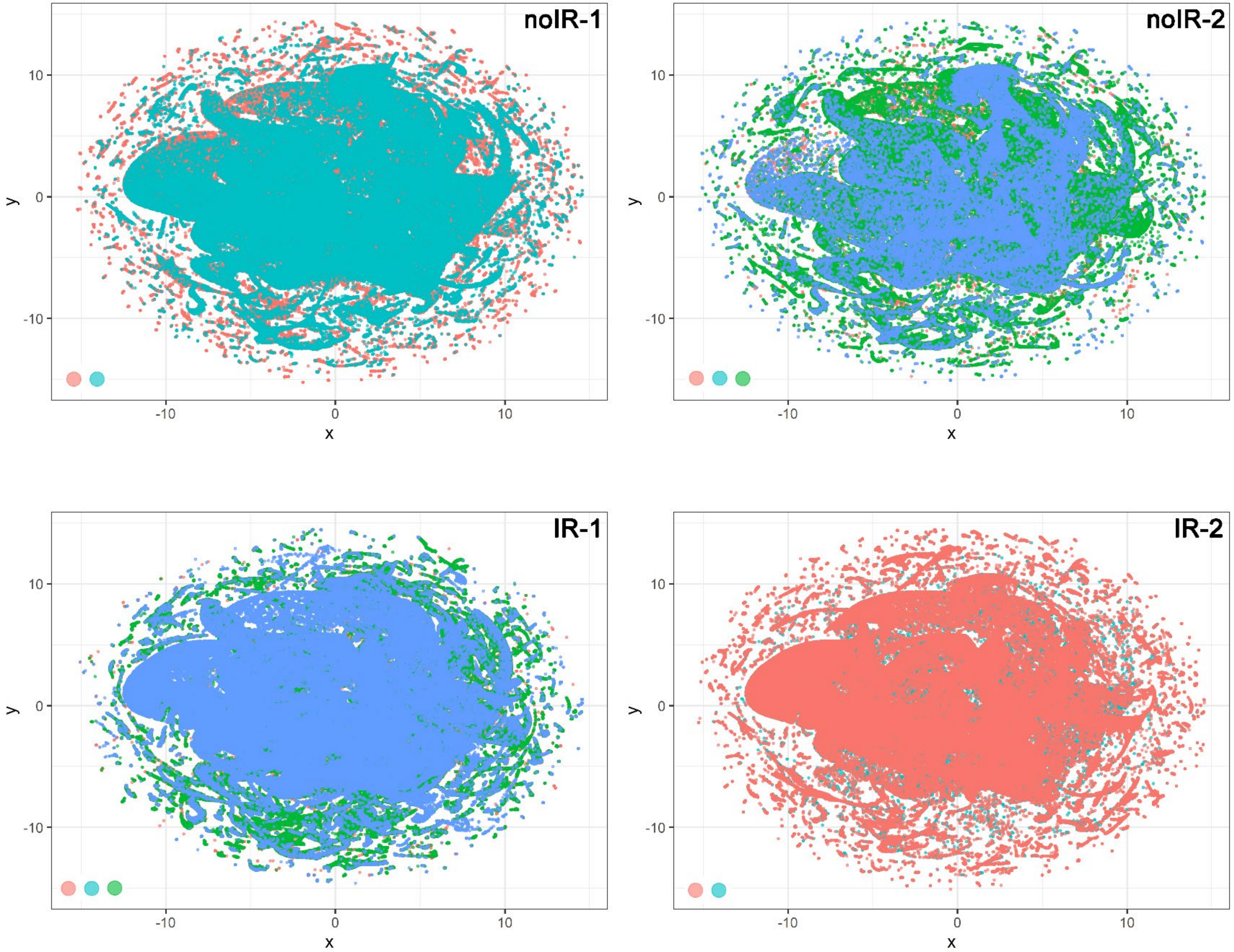

Supplemental Figure S14. UMAP analysis of all per pixel concentrations for all interspersed cell tissue regions from all animals. For ROI generation see Supplemental Figures 3-6. Individual interspersed cell regions are represented by different color dots.
