# Supplementary material for: X-ray Fluorescence Microscopy to Develop Elemental Classifiers and Investigate Elemental Signatures in BALB/c Mouse Intestine a Week after Exposure to 8 Gy of Gamma Rays
Source: Int J Mol Sci. 2024 Sep 24;25(19):10256. doi: 10.3390/ijms251910256 (PMC11477073; doi:10.3390/ijms251910256)
Supplement: Supplementary file 1 [file ijms-25-10256-s001.zip › SupplementalTables.pdf]

Supplemental Tables

X-ray Fluorescence Imaging to Develop Elemental Classifiers and Investigate Elemental Signatures in BALB/c Mouse Intestine a Week after Exposure to 8 Gy of Gamma Rays

Anthony Smith[1], Katrina Dobinda[1], Si Chen[2], Peter Zieba[1], Tatjana Paunesku[1], Zequn Sun[1,*] and Gayle Woloschak[1,*]

[1] Feinberg School of Medicine, Northwestern University
[2] X-ray Imaging Division, Advanced Photon Source, Argonne National Laboratory
[*] Co-corresponding authors

Supplemental Table S1. Mean per Pixel Elemental Concentrations for Specific Subcellular Regions for Specific Cell Groups in Different Animals with (Standard Deviation)

			Per Pixel Element Concentration (micrograms/cm^2)					
Cell Group	Animal	Subcellular Region	P	S	Ca	Fe	Cu	Zn
Crypt Cells	noIR-1	Nucleus	5.37 (1.33)	2.57 (0.59)	0.31 (0.09)	0.06 (0.02)	0.01 (0.0041)	0.06 (0.01)
Crypt Cells	noIR-2	Nucleus	5.87 (1.81)	2.32 (0.57)	0.25 (0.09)	0.03 (0.01)	0.01 (0.0061)	0.02 (0.0077)
Crypt Cells	IR-1	Nucleus	3.1 (1.13)	1.62 (0.53)	0.23 (0.09)	0.03 (0.02)	0.0052 (0.0046)	0.02 (0.0094)
Crypt Cells	IR-2	Nucleus	4.22 (1.3)	2.27 (0.55)	0.34 (0.1)	0.04 (0.02)	0.0057 (0.0035)	0.04 (0.01)
Interspersed Cells	noIR-1	Cytosol	1.93 (1.26)	2.41 (0.79)	0.14 (0.07)	0.04 (0.03)	0.0084 (0.0034)	0.03 (0.01)
Interspersed Cells	noIR-1	Nucleus	5.63 (1.84)	2.41 (0.63)	0.29 (0.1)	0.05 (0.02)	0.01 (0.0042)	0.05 (0.01)
Interspersed Cells	noIR-2	Cytosol	1.95 (1.46)	1.32 (0.57)	0.09 (0.06)	0.02 (0.02)	0.0062 (0.0047)	0.01 (0.01)
Interspersed Cells	noIR-2	Nucleus	4.24 (1.68)	1.44 (0.47)	0.16 (0.08)	0.03 (0.03)	0.0065 (0.0038)	0.01 (0.0055)
Interspersed Cells	IR-1	Cytosol	1.65 (1.38)	1.37 (0.72)	0.14 (0.1)	0.06 (0.13)	0.005 (0.0044)	0.02 (0.0097)
Interspersed Cells	IR-1	Nucleus	3.46 (1.62)	1.42 (0.57)	0.22 (0.1)	0.04 (0.04)	0.0051 (0.0056)	0.02 (0.0096)
Interspersed Cells	IR-2	Cytosol	1.63 (1.05)	3.31 (1.41)	0.2 (0.1)	0.06 (0.05)	0.0065 (0.004)	0.03 (0.02)
Interspersed Cells	IR-2	Nucleus	4.29 (1.69)	3.14 (1.02)	0.31 (0.12)	0.05 (0.03)	0.0063 (0.0037)	0.04 (0.01)
Villus Cells	noIR-1	Cytosol	1.83 (0.75)	2.57 (0.94)	0.12 (0.05)	0.04 (0.02)	0.008 (0.0042)	0.03 (0.0084)
Villus Cells	noIR-1	Nucleus	3.6 (1.2)	1.95 (0.59)	0.22 (0.08)	0.04 (0.02)	0.0094 (0.0038)	0.04 (0.01)
Villus Cells	noIR-2	Cytosol	1.53 (0.49)	3.23 (0.65)	0.1 (0.04)	0.07 (0.07)	0.0073 (0.0029)	0.04 (0.0092)

			Per Pixel Element Concentration (micrograms/cm^2)					
Cell Group	Animal	Subcellular Region	P	S	Ca	Fe	Cu	Zn
Villus Cells	noIR-2	Nucleus	3.63 (1.32)	2.32 (0.43)	0.22 (0.06)	0.06 (0.01)	0.0079 (0.0029)	0.06 (0.009)
Villus Cells	IR-1	Cytosol	1.83 (0.88)	2.04 (0.77)	0.16 (0.07)	0.04 (0.03)	0.0064 (0.0053)	0.03 (0.01)
Villus Cells	IR-1	Nucleus	3.51 (1.56)	1.79 (0.67)	0.21 (0.1)	0.04 (0.02)	0.006 (0.0051)	0.03 (0.01)
Villus Cells	IR-2	Cytosol	4.2 (1.95)	3.62 (1.17)	0.4 (0.19)	0.04 (0.02)	0.0083 (0.0047)	0.06 (0.02)
Villus Cells	IR-2	Nucleus	7.17 (2.25)	3.8 (0.87)	0.53 (0.16)	0.05 (0.02)	0.0082 (0.0043)	0.07 (0.02)

Supplemental Table S2. All Anova P-Values for Comparisons for Element per Pixel Concentration within Different Cell Types (Interspersed Cells - IC, Crypt Cells - CC and Villus Cells - VC) and Subcellular Regions within Same Animals

		Cell Type		
Animal	Element	IC	CC	VC
noIR-1	P	<0.001	<0.001	<0.001
noIR-1	S	<0.001	<0.001	<0.001
noIR-1	Ca	<0.001	<0.001	<0.001
noIR-1	Fe	<0.001	<0.001	<0.001
noIR-1	Cu	<0.001	<0.001	<0.001
noIR-1	Zn	<0.001	<0.001	<0.001
noIR-2	P	<0.001	<0.001	<0.001
noIR-2	S	<0.001	<0.001	<0.001
noIR-2	Ca	<0.001	<0.001	<0.001
noIR-2	Fe	<0.001	<0.001	<0.001
noIR-2	Cu	<0.001	<0.001	<0.001
noIR-2	Zn	<0.001	<0.001	<0.001
IR-1	P	<0.001	<0.001	<0.001
IR-1	S	<0.001	<0.001	<0.001
IR-1	Ca	<0.001	<0.001	<0.001
IR-1	Fe	<0.001	<0.001	<0.001
IR-1	Cu	<0.001	<0.001	<0.001
IR-1	Zn	<0.001	<0.001	<0.001
IR-2	P	<0.001	<0.001	<0.001
IR-2	S	<0.001	<0.001	<0.001
IR-2	Ca	<0.001	<0.001	<0.001
IR-2	Fe	<0.001	<0.001	<0.001
IR-2	Cu	<0.001	<0.001	<0.001
IR-2	Zn	<0.001	<0.001	<0.001

Supplemental Table S3. Anova P - Values for Comparisons for Different Cell Types (Interspersed Cells - IC, Crypt Cells - CC and Villus Cells - VC) and Subcellular Regions within Same Animals Ignoring Individual Elements

	Cell Type		
Animal	IC	CC	VC
noIR-1	<0.001	<0.001	<0.001
noIR-2	<0.001	<0.001	<0.001
IR-1	<0.001	<0.001	<0.001
IR-2	<0.001	<0.001	<0.001

Supplemental Table S4. Mean per Pixel Elemental Concentrations for Identical Subcellular Regions of Identical Cells within a Cell Group Acquired by Regular or Undersampling Scans with (Standard Deviation)

			Per Pixel Element Concentration (microgram/cm^2)					
Cell Group	Scan Type	Subcellular Region	P	S	Ca	Fe	Cu	Zn
Crypt Cells	Undersampling	Nucleus	4.26 (1.24)	2.35 (0.57)	0.37 (0.11)	0.04 (0.02)	0.0063 (0.0037)	0.05 (0.01)
Crypt Cells	Regular Scan	Nucleus	4.41 (1.09)	2.28 (0.48)	0.37 (0.1)	0.04 (0.01)	0.0064 (0.0029)	0.05 (0.01)
Interspersed Cells	Undersampling	Cytosol	1.22 (0.87)	3.26 (1.05)	0.14 (0.07)	0.07 (0.08)	0.0069 (0.0041)	0.03 (0.01)
Interspersed Cells	Regular Scan	Cytosol	1.27 (0.72)	3.5 (0.96)	0.15 (0.05)	0.08 (0.09)	0.0071 (0.003)	0.03 (0.01)
Interspersed Cells	Undersampling	Nucleus	4.19 (1.88)	3.47 (0.78)	0.28 (0.1)	0.07 (0.03)	0.0074 (0.004)	0.04 (0.01)
Interspersed Cells	Regular Scan	Nucleus	4.34 (1.79)	3.24 (0.75)	0.28 (0.09)	0.07 (0.03)	0.007 (0.0029)	0.04 (0.01)

Supplemental Table S5. T-Test P-Values for Comparisons for Identical Cells' Subcellular Regions between Regular and Undersampled Scans

	Per Pixel Element Concentration (microgram/cm^2)					
Specific Cell ID	P	S	Ca	Fe	Cu	Zn
CC 3 Nucleus	<0.001	0.18	0.24	<0.001	0.18	<0.001
CC 4 Nucleus	<0.001	0.17	<0.001	0.05	0.97	<0.001
CC 5 Nucleus	<0.001	0.09	<0.001	0.2	0.18	<0.001
CC 6 Nucleus	<0.001	<0.001	<0.001	<0.001	<0.001	<0.001
CC 8 Nucleus	0.02	0.47	0.82	0.42	0.18	0.98
CC 9 Nucleus	<0.001	0.4	<0.001	0.002	0.009	0.002
IC 1 Nucleus	0.2	0.99	0.17	0.04	0.26	0.08
IC 1 Cytosol	<0.001	<0.001	<0.001	<0.001	<0.001	<0.001
IC 4 Nucleus	0.05	<0.001	0.84	0.19	0.47	0.05
IC 4 Cytosol	<0.001	0.04	<0.001	0.07	0.96	0.06
IC 5 Nucleus	<0.001	0.24	<0.001	0.03	0.6	0.001
IC 5 Cytosol	<0.001	<0.001	<0.001	0.19	0.06	<0.001
IC 6 Nucleus	0.05	0.61	0.14	0.18	0.61	0.001
IC 6 Cytosol	0.13	0.05	0.61	0.03	0.33	0.61
IC 7 Nucleus	<0.001	<0.001	<0.001	<0.001	<0.001	<0.001
IC 7 Cytosol	<0.001	<0.001	<0.001	<0.001	<0.001	<0.001

Supplemental Table S6. T-Test P-Values Expressed as “Same” or “Different” for p >=0.05 or p<0.05 (per Supplemental Table S5.), Respectively

Cell ID	P	S	Ca	Fe	Cu	Zn
CC 3 Nucleus	Different	Same	Same	Different	Same	Different
CC 4 Nucleus	Different	Same	Different	Different	Same	Different
CC 5 Nucleus	Different	Same	Different	Same	Same	Different
CC 6 Nucleus	Different	Different	Different	Different	Different	Different
CC 8 Nucleus	Different	Same	Same	Same	Same	Same
CC 9 Nucleus	Different	Same	Different	Different	Different	Different
IC 1 Nucleus	Same	Same	Same	Different	Same	Same
IC 1 Cytosol	Different	Different	Different	Different	Different	Different
IC 4 Nucleus	Same	Different	Same	Same	Same	Different
IC 4 Cytosol	Different	Different	Different	Same	Same	Same
IC 5 Nucleus	Different	Same	Different	Different	Same	Different
IC 5 Cytosol	Different	Different	Different	Same	Same	Different
IC 6 Nucleus	Different	Same	Same	Same	Same	Different
IC 6 Cytosol	Same	Different	Same	Different	Same	Same
IC 7 Nucleus	Different	Different	Different	Different	Different	Different
IC 7 Cytosol	Different	Different	Different	Different	Different	Different

Supplemental Table S7. Absolute Percent Differences for Elemental per Pixel Concentrations for Identical Cells' Subcellular Regions in Regular and Undersampled Scans

	Element					
Specific Cell ID	P	S	Ca	Fe	Cu	Zn
CC 3 Nucleus	0.0464	0.0020	0.0089	0.0577	0.0267	0.0446
CC 4 Nucleus	0.0889	0.0189	0.0597	0.0188	0.0511	0.0370
CC 5 Nucleus	0.0609	0.0086	0.0353	0.0234	0.0133	0.0266
CC 6 Nucleus	0.1075	0.1711	0.1773	0.2024	0.1361	0.1782
CC 8 Nucleus	0.0500	0.0046	0.0139	0.0247	0.0512	0.0016
CC 9 Nucleus	0.1573	0.0137	0.0976	0.0415	0.2968	0.0202
IC 1 Nucleus	0.0395	0.0336	0.0804	0.0298	0.0663	0.0671
IC 1 Cytosol	0.1930	0.1667	0.1646	0.1431	0.1709	0.1206
IC 4 Nucleus	0.0789	0.0901	0.0278	0.0266	0.0286	0.0135
IC 4 Cytosol	0.0430	0.0408	0.0766	0.0547	0.0664	0.0025
IC 5 Nucleus	0.1509	0.0250	0.1594	0.0657	0.0624	0.0594
IC 5 Cytosol	0.1202	0.0580	0.1117	0.0043	0.0580	0.1158
IC 6 Nucleus	0.0653	0.0046	0.0729	0.0590	0.0516	0.1433
IC 6 Cytosol	0.0664	0.0305	0.0142	0.0264	0.0696	0.0472
IC 7 Nucleus	0.2151	0.2064	0.1934	0.1679	0.1004	0.1585
IC 7 Cytosol	0.1352	0.1058	0.1148	0.1749	0.0948	0.0967

Supplemental Table S8. T-Test P-Values Expressed as Differences (per Supplemental Table S6.) after Application of 5% Cutoff for Absolute Percent Differences (per Supplemental Table S7.)

Cell ID	P	S	Ca	Fe	Cu	Zn
CC 3 Nucleus	Same	Same	Same	Different	Same	Same
CC 4 Nucleus	Different	Same	Different	Same	Same	Same
CC 5 Nucleus	Different	Same	Same	Same	Same	Same
CC 6 Nucleus	Different	Different	Different	Different	Different	Different
CC 8 Nucleus	Same	Same	Same	Same	Same	Same
CC 9 Nucleus	Different	Same	Different	Same	Different	Same
IC 1 Nucleus	Same	Same	Same	Same	Same	Same
IC 1 Cytosol	Different	Different	Different	Different	Different	Different
IC 4 Nucleus	Same	Different	Same	Same	Same	Same
IC 4 Cytosol	Same	Same	Different	Same	Same	Same
IC 5 Nucleus	Different	Same	Different	Different	Same	Different
IC 5 Cytosol	Different	Different	Different	Same	Same	Different
IC 6 Nucleus	Different	Same	Same	Same	Same	Different
IC 6 Cytosol	Same	Same	Same	Same	Same	Same
IC 7 Nucleus	Different	Different	Different	Different	Different	Different
IC 7 Cytosol	Different	Different	Different	Different	Different	Different

Supplemental Table S9. Mean Elemental per Pixel Concentrations for Tissue Region Data from Different Animals with (Standard Deviation)

		Per Pixel Element Concentration (micrograms/cm^2)					
Animal	Tissue Region	P	S	Ca	Fe	Cu	Zn
noIR-1	Crypt Area	4.09 (1.76)	2.95 (1.36)	0.3 (0.17)	0.06 (0.03)	0.01 (0.0042)	0.05 (0.02)
noIR-1	Interspersed Cells Area	2.1 (2.03)	1.77 (1.06)	0.15 (0.14)	0.04 (0.03)	0.0072 (0.0052)	0.03 (0.02)
noIR-2	Crypt Area	4.26 (1.97)	2.43 (1.1)	0.2 (0.11)	0.04 (0.03)	0.0097 (0.0073)	0.02 (0.01)
noIR-2	Interspersed Cells Area	2.1 (1.88)	1.4 (0.88)	0.1 (0.1)	0.02 (0.03)	0.0065 (0.0071)	0.01 (0.01)
IR-1	Crypt Area	2.45 (1.38)	1.99 (1.11)	0.2 (0.1)	0.04 (0.03)	0.0057 (0.0052)	0.02 (0.01)
IR-1	Interspersed Cells Area	1.81 (3.15)	1.64 (1.4)	0.14 (0.1)	0.06 (0.13)	0.0056 (0.0093)	0.02 (0.02)
IR-2	Crypt Area	3.32 (1.51)	2.26 (0.65)	0.29 (0.11)	0.04 (0.02)	0.0059 (0.0029)	0.04 (0.01)
IR-2	Interspersed Cells Area	1.8 (1.88)	2.29 (1.79)	0.17 (0.15)	0.04 (0.07)	0.0055 (0.004)	0.03 (0.02)

Supplemental Table S10. Anderson Darling Test P-Values for Elemental per Pixel Concentrations Comparing Crypt Regions vs. Interspersed Cells Tissue Regions in Different Animals

	Element					
animal	P	S	Ca	Fe	Cu	Zn
noIR-1	<0.001	<0.001	<0.001	<0.001	<0.001	<0.001
noIR-2	<0.001	<0.001	<0.001	<0.001	<0.001	<0.001
IR-1	<0.001	<0.001	<0.001	<0.001	<0.001	<0.001
IR-2	<0.001	<0.001	<0.001	<0.001	<0.001	<0.001
